# Squamate bone taphonomy: A new experimental framework and its application to the Natufian zooarchaeological record

**DOI:** 10.1038/s41598-020-66301-5

**Published:** 2020-06-10

**Authors:** Ma‘ayan Lev, Mina Weinstein-Evron, Reuven Yeshurun

**Affiliations:** Zinman Institute of Archaeology, University of Haifa, 199 Abba-Khoushy Ave., Haifa, 3498838 Israel

**Keywords:** Archaeology, Palaeoecology

## Abstract

Squamate (lizard and snake) remains are abundant in the terminal Pleistocene Natufian archaeological sites of the Levant, raising the question of whether they constitute part of the broad-spectrum diet characteristic of this period. However, the role of squamates in Natufian diets remains unclear, as they are taphonomically under-studied. We conducted a series of experiments and actualistic observations that tested the impact of pre- and post-depositional processes on squamate vertebrae. We emphasized the multiple destruction processes that leave overlapping or altered marks on the bones, such as digestion marks that were modified by trampling. The resulting bone modification typology provides a tool for studying archaeological squamate remains. The experimental data were compared to the archaeological bone samples of the Natufian sequence of el-Wad Terrace (Mount Carmel, Israel, 15,000–12,000 cal BP). The Natufian squamate samples deviate from all actualistic ones in their lesser evidence of digestion and much greater indications for trampling, erosion and breakage. The taphonomic study, coupled with intra-site analysis, has unraveled the complex depositional history of el-Wad Terrace, enabling us to differentiate between cultural and non-cultural contexts and to identify possible human consumption of the European glass lizard and the large whip snake in the Natufian.

## Introduction

**Figure 1:**
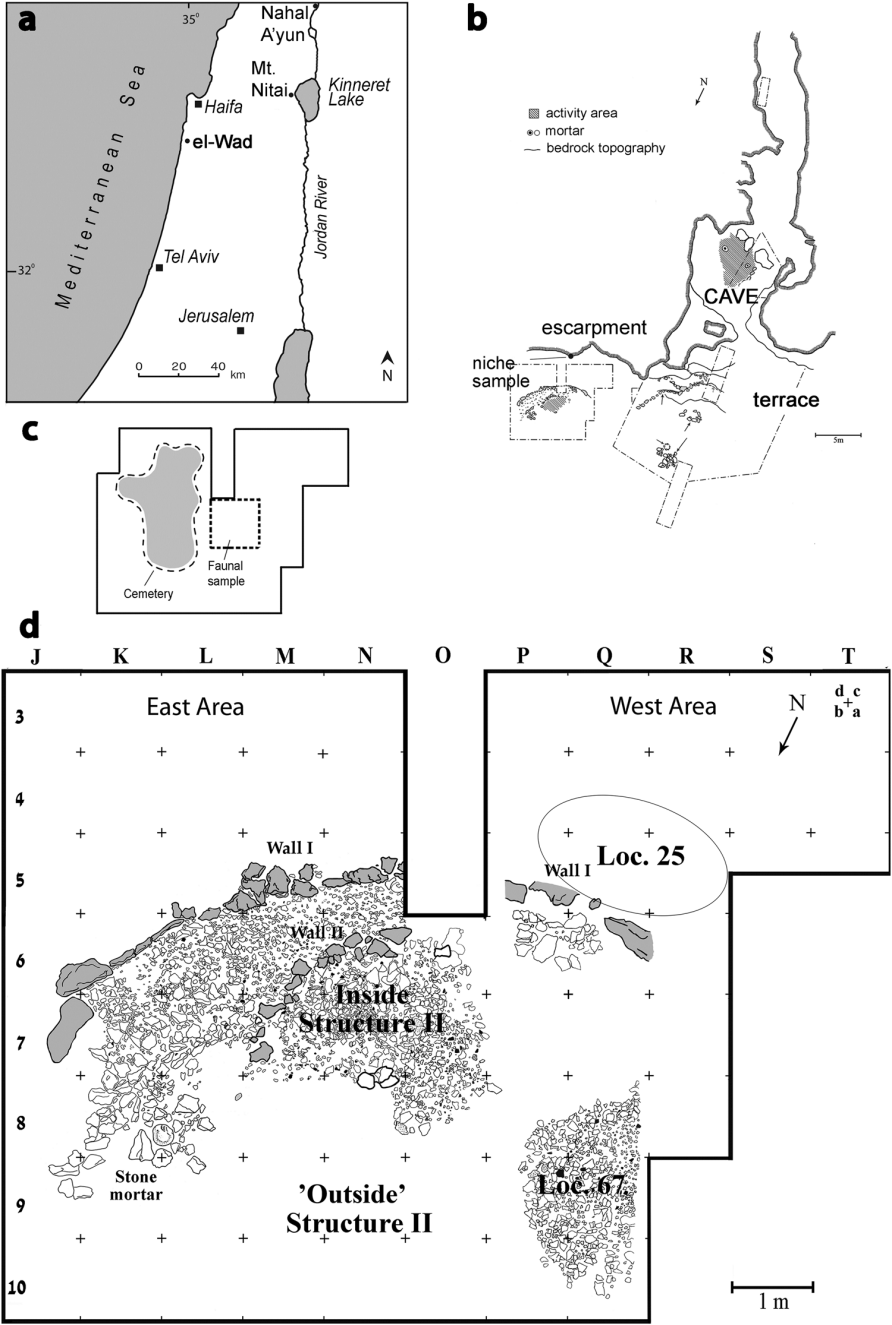
Provenance of the archaeological squamates assemblage: (**a**) Location map showing the Natufian site of el-Wad, Israel and the location of eagle-owl pellets Nahal ‘Ayun and Mount Nitai; (**b**) plan of el-Wad cave, adjacent terrace and the location of the niche sample; (**c**) plan of the NE Terrace excavation - the Late Natufian layer; (**d**) plan of the terrace excavation of the Early Natufian layer with the different contexts presented in this study.

Squamate (lizard and snake) remains have been sporadically studied in zooarchaeology^1–12^, and rarely has their role in human subsistence been established (but see^8^). These remains are known to have been abundant in the early sedentary hamlets of the Natufian culture of the Levant^13^ (late Epipaleolithic, ca. 15,000–11,700 cal BP), together with abundant small game remains and naturally-deposited micro-mammals. This raises the question of squamate accumulation mechanisms and especially whether they were part of the broad human subsistence that characterized this period^13–16^. The site of el-Wad Terrace (EWT), Mount Carmel, Israel (Fig. 1), one of the major Natufian hamlets of the Levantine Mediterranean region, includes both the Early Natufian (EN) and the Late Natufian (LN) phases, the former characterized by numerous architectural sub-phases^17,18^. Recent excavations at the site have exposed dense layers with extremely rich lithic and faunal assemblages that include a high percentage of squamate remains^13^.

The expansion of diet breadth to include a wide variety of game, often referred to as the Broad Spectrum Revolution (BSR^19^), which arguably marks considerable resource intensification, is a prominent topic in Natufian studies. Broadening Natufian diets may signal an early shift to sedentism among the foraging groups of the terminal Pleistocene Levant^13,20–24^. This process could be instrumental in the Neolithization process that immediately succeeded the Natufian^25^. While squamates are abundant at many Natufian sites and at EWT in particular, they are usually excluded from human subsistence studies that attempt to establish diet breadth by game diversity and prey ranking. For the most part, they have not benefitted from comprehensive taphonomic studies to establish their origin (but see^26^). However, the abundance of squamate remains in the Natufian (one third to one half of the NISP^13^) may suggest that they played a role in the expansion of diet breadth.

Detailed taphonomic studies regarding bone accumulation and post-depositional processes of squamates are needed to test the hypothesis that some of their remains constitute food refuse of broad-diet Natufian communities, rather than animal predation or natural death. However, detailed taphonomic studies regarding herpetofauna and most specifically squamates are rare and are mostly focused on reptile and amphibian long bones and mandibles^27^, whereas the vast majority of our zooarchaeological record is composed of vertebrae. Some studies describe digestion marks and other bone-surface modifications on squamates (e.g.^9,10,28^) but they were not based on clear experimental taphonomic data. These studies contain neither detailed descriptions of the impact of other post-depositional processes such as weathering, trampling and abrasion on squamates vertebrae, nor explain how overlapping processes can be discerned in a complex taphonomic system.

To overcome the gap in taphonomic frames of reference we experimentally tested four post-depositional processes: weathering, burning (which, in the Natufian context of EWT, mostly represents unintentional charring of preexisting refuse^29^), sediment erosion and trampling on three bone accumulations: fresh European glass lizard (*Pseudopods apodus*) and common viper (*Vipera palaestinae*) carcasses, and digested squamate bones from eagle owl (*Bubo bubo*) pellets.

Following the experiments, bone-surface modifications and fragmentation patterns on the squamate vertebrae were defined and categorized, with the aim of creating a typology of bone modifications that may be used to discern different accumulation and modification agents.

Subsequently, the taphonomic markers identified in the experimental material were compared to the Natufian squamate assemblage of EWT. Together with other variables such as vertebra size, species abundance and intra-site distribution, we managed to reconstruct the complex taphonomic history of the remains and to differentiate between accumulation and post-depositional bone surface modification at the site. Furthermore, through the detailed taphonomic analysis we were able to differentiate between cultural and non-cultural contexts, with the former suggesting human consumption of the European glass lizard and the large whip snake.

## Results

### Experimental results

During our weathering experiment, observed for a period of 12 months, the glass lizard carcass remained in complete articulation with scales for the first two months (November and December, 2018), showing no major changes; descaling subsequently occurred but the skeleton remained in complete articulation. After three months the skeleton started to disarticulate, with some bones separated from the main body of the lizard. The most drastic change occurred after five months when most of the skeleton was disarticulated. After nine months in the field the bones were mostly disarticulated (Supplementary Fig. S1). Only very slight cracking of the ventral surface of the glass lizard vertebrae appeared after nine months, while the digested bones showed no change at all. After one year the cracking of the glass lizard vertebrae ventral surfaces progressed to Andrews’ stage 1 (Supplementary Table S1). No bone surface modifications (e.g. cracking) were observed on the digested bones at any point in the experiment. The experiment is ongoing and is now in its second year.

Our burning experiment produced cracking of bone surfaces, mainly on the condyle, cotyle, parapophysis and diapophysis of vertebrae (Supplementary Fig. S2). Greater burning intensity (time × temperature) positively correlated with more extreme cracking (Spearman’s r = 0.97 p < 0.01). Natural and digested bones were found to have been similarly affected in each time/temperature combination (Supplementary Table S2, Supplementary Fig. S3). Squamate vertebrae appear to turn black in lower temperatures than fish cranial remains^30^ and macro-mammal bones^31,32^ perhaps due to their smaller size and larger surface area. Owl digestive process does not appear to have an impact on the bone charring temperature.

Erosion by sediment produced diagnostic marks on the diapophysis, parapophysis and condyle to varying degrees. On untreated bones the slightest erosion was observed as slight abrasion of the bone surface. Moderate erosion occurred as abrasion of the bone surface that produced slight perforations with irregular edges on the protruding edges of the diapophysis, parapophysis and on the condyle. Great erosion occurred as large perforations with irregular edges on the entire surface of the diapophysis, parapophysis and on the condyle, and extreme erosion occurred as complete abrasion of the diapophysis and the parapophysis and large perforations with irregular edges on the condyle. On digested bones slight erosion was observed as slight abrasion of the digested bone surface and smoothing of the regular-edge perforation on the protruding edges of the diapophysis and parapophysis. Moderate erosion occurred as the abrasion smoothed the regular-edge perforation on the entire surface of the diapophysis, parapophysis and condyle. Great erosion occurred as parts of the diapophysis and parapophysis were broken and rounded along with the smoothing of the bone surface and, like the untreated bones, extreme erosion occurred as complete abrasion of the diapophysis and the parapophysis (Supplementary Fig. S4).

Overall, longer rotation time led to greater erosion of the bone surface (Supplementary Table 3; χ^2^ = 3.85, p = 0.05). Some variability was observed according to the state of the bone (untreated, digested or burnt). In the untreated sub-sample, the longer the erosion time the more abraded the bones surface became. Burnt bones were found to be more susceptible to erosion, with extreme erosion in three out of seven specimens. The digested bones produced variable erosion irrespective of time.

Trampling similarly produced abrasion of the bone surfaces and perforation with irregular edges on the diapophysis, the parapophysis and on the condyle (Supplementary Fig. S5). Trampling also produced breakage of the protruding parts of the vertebrae (diapophysis and parapophysis, prezygapophysis, prezygapophyseal articular facet, hypapophysis and postzygapophyses), which we scored from 0 (all the protruding parts were complete) to 7 (all the protruding parts were broken). Here too, the state of the bone (untreated, digested or burnt) appeared to have had an impact. Untreated and digested bones were rarely broken, while burnt bones had a noticeably lower completeness index and higher protruding-part breakage index: of 17 burnt specimens, one was not broken at all, three had a low breakage index, six had a moderate breakage index, four had a high breakage index and three were broken beyond retrieval (Supplementary Table S4, Supplementary Fig. S6). Our pilot erosion-by-sediment and trampling experiments revealed the overall impact that these processes would have had on squamate vertebrae, but further experimentation is needed to verify the extent of variability among the untreated, digested and burnt bones.

Our actualistic observations included eagle owl pellets collected from two locations in northern Israel, Mount Nitai and Nahal ‘Ayun. Indicative digestion marks were found to varying degrees in both eagle owl pellets accumulations. The marks consist of perforations of the bone surface with regular edges on the condyle, cotyle, diapophysis and parapophysis (Supplementary Fig. S7). Of the specimens in the pellets, 81% displayed these marks; of those, half were of low intensity, 22% were of moderate intensity and high intensity was very rare (1%, n = 4 of 152 in total) (Supplementary Table S5). Breakage patterns showed that the vertebrae in the pellets were mostly whole or only slightly broken (complete = 74%, slight = 25%) with one vertebra with moderate breakage intensity (1%).

In the presumed owl pellet remains retrieved from a niche in the cliff overhanging the el-Wad Terrace by L. Weissbrod^33^, henceforth el-Wad Niche, the previously identified indicative digestion marks (perforation of the bone surface with regular edges) were abundant (51%, n = 138 of total 273). Thirty-three percent (n = 89) were of low intensity, 12% (n = 33) were of moderate intensity and, like the eagle owl pellets, high intensity was rare and appeared only on 5% (n = 13) of the specimens (Supplementary Table S6). In addition, perforations with irregular edges, which in our experiment had resulted from erosion by sediment and trampling, were observed in varying intensity; most of the assemblage (53% n = 142 of total 268) had slight to moderate erosion intensity, great intensity was less common (11% n = 29) and extreme intensity was very rare (1% n = 2). Breakage patterns showed that the vertebrae of the assemblage were mostly whole or only slightly broken (complete = 27%, slight = 38%) but moderate (19%) great (10%) and extreme (6%) breakage intensities were also noted (Supplementary Table S7).

### Typology of bone surface modifications on squamate vertebrae

**Figure 2:**
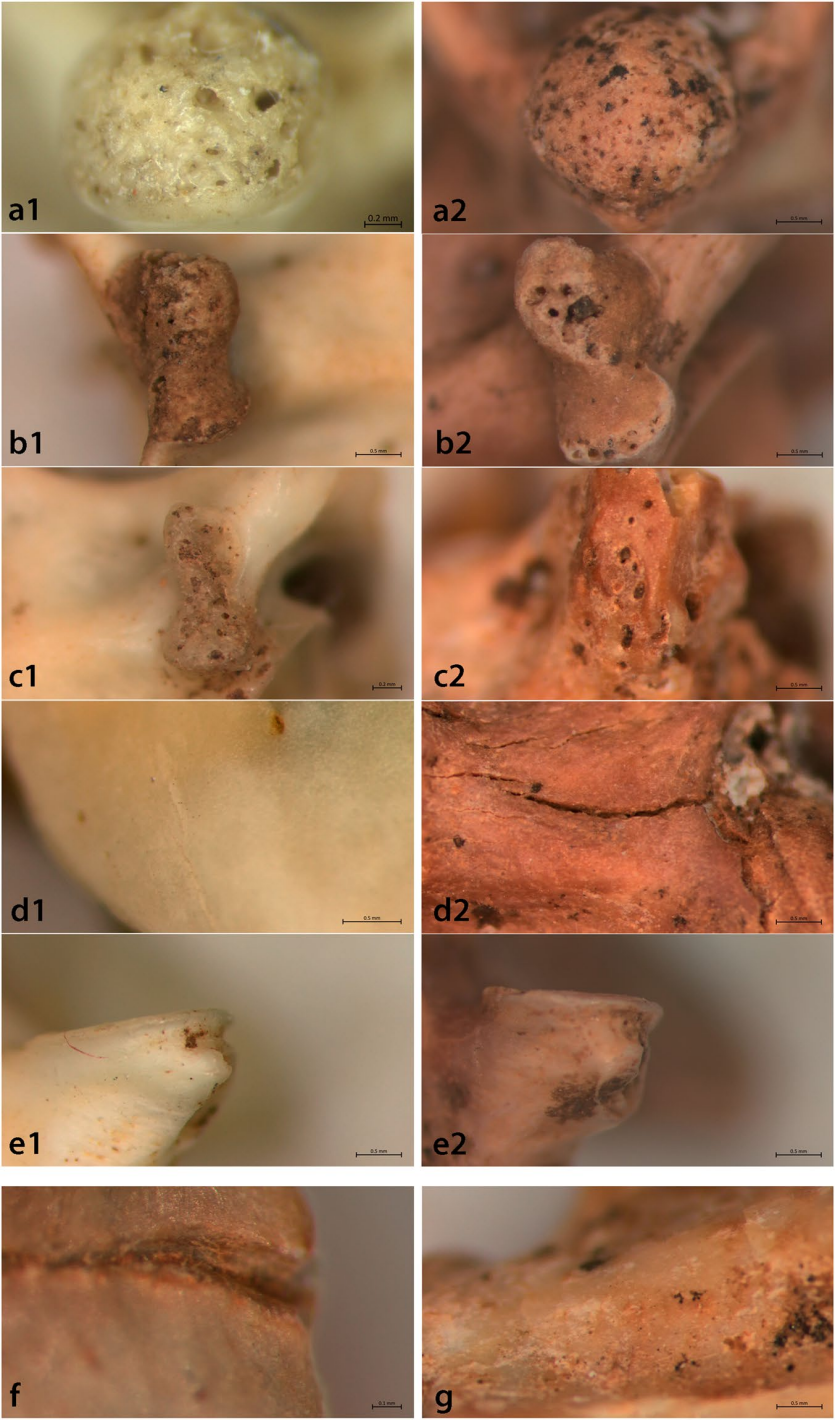
Bone modification types in the experimental material (left column) and their equivalents in the archaeological assemblage (right column). (**a**) Regular-edge perforations from the Mount Nitai pellet; (**b**) irregular-edge perforations from the trampling experiment; (**c**) smoothing of the regular-edge perforation from the trampling experiment; (**d**) cracking of the bone surface from the weathering experiment; (**e**) breakage of protruding parts of the vertebrae from the trampling experiment; (**f**) linear striations from the archaeological sample; (**g**) flaking of the bone surface from the archaeological sample.

Following the experiments and observations on the pellets, we identified five bone surface modification types and linked them to different accumulation and modification scenarios, as follows:

Type A. Perforation of the bone surface with regular edges on the condyle, cotyle, diapophysis and parapophysis along with smoothing and polishing of the bone surface (Fig. 2a). Similar marks were identified previously on squamate bones^10^ and on micro-mammals (e.g.^34,35^) and were attributed to digestion. Our observations confirm their assignment to digestion, as they were observed only in the pellets and in none of the other experimental sets.

Type B. Perforations with irregular edges on the bone surface that were located on the condyle, diapophysis and parapophysis and rarely on the cotyle (Fig. 2b). We produced this modification type by the erosion-by-sediment and trampling experiments. Type B was not found in any of the fresh eagle owl pellets, but was found in 45% of the specimens of el-Wad Niche, mostly to a slight degree (20.6% of the total assemblage). Owls trampling in their roost and fluvial transport may be the sources of such impact in the Niche assemblage^33^.

Type C. Smoothing of the regular-edge perforation (Type A) (Fig. 2c). This process was observed when digested bones were subjected to erosion by sediment and trampling (see Supplementary Figs. S4, S5).

Type D. Cracking, splits and cracks that penetrate the surface of the bone but do not cause loss of surface tissue^35^ were observed on the bone surface on the condyle, cotyle, diapophysis and parapophysis (Fig. 2d). We observed this modification in the burning experiment. In addition, slight cracking of the ventral surface of the vertebrae occurred in the advanced stages of our weathering experiment.

Type E. Breakage of protruding parts of the vertebrae (diapophysis and parapophysis, prezygapophysis, prezygapophyseal articular facet, hypapophysis and postzygapophyses). This damage was produced in the erosion-by-sediment and trampling experiments on untreated, digested and burnt bones (Fig. 2e). Weathering, and burning per se, did not create this damage.

In addition, two modification types, which were observed on our archaeological material (see below) but not in our experimental dataset, should be mentioned:

Type F. linear striations with a v-shaped cross section on the lateral left postzygapophysis (from posterior view; Fig. 2f).

Type G. Flaking, the loss of the bone surface tissue, described for mammal bones by Fernandez-Jalvo and Andrews^34^ as the result of weathering and mammalian and raptor digestion) (Fig. 2g). Our experiments did not replicate this modification.

### The archaeological case study

The EN of EWT yielded the NISP of 2992 squamate remains, from a sample of 9146 archaeofaunal remains (33%); the 1-mm sample added 599 NISP. The LN sample yielded the NISP of 939 squamate remains, from a sample of 2419 archaeofaunal remains (39%); the 1-mm sample added 539 NISP. The squamates of the assemblage are represented mainly by vertebrae (95% of the total assemblage), supplemented by some skull pieces. Of the total NISP, 2041 specimens were identified to the species level and the rest to family or to sub-order (Table 1). To prevent size biases only excavation units (baskets) that underwent both 5-mm and 1-mm collection were used for taxonomic evenness and vertebra size comparisons (Supplementary Table S8).

**Table 1:** Taxonomic composition of the el-Wad Terrace total squamate assemblages by context (NISP).

Species	Inside	Outside	L. 67 area	L. 25	LN
**Lizard**					
European glass lizard (*Pseudopus apodus*)	263	185	150	12	135
Roughtail rock agama (*Stellagama stellio ssp*.)	13	20	13	8	32
Schneider’s skink (*Eumeces schneideri pavimentatus*)	0	3	0	0	6
Levant green lizard (*Lacerta media israelica*)	1	0	1	0	3
Common chameleon (*Chamaeleo chamaeleon recticrista*)		1	0	0	1
Scincidae	1	0	0	0	2
Lacertidae	0	1	0	1	2
Other lizards	13	13	9	5	38
**Snake**					
Large whip snake (*Dolichophis jugularis*)	208	199	88	21	119
Eastern Montpellier snake (*Malpolon insignitus*)	137	139	44	19	101
Coin-marked snake (*Hemorrhois nummifer*)	7	5	1		1
Common viper (*Daboia palaestinae*)	26	22	8	5	35
Javelin sand boa (*Eryx jaculus*)	0	0	0	6	1
Dice snake (*Natrix tessellata*)	0	0	0	0	1
Colubridae-Colubrinae	52	40	23	5	75
Colubridae- Psamophiininae	12	6	1	3	1
Colubridae-Elaphe	0	0	1	0	0
Colubridae	157	143	64	38	232
Other snakes	611	420	265	101	693
Total number of NISP	1501	1197	668	224	1478
**Species only**					
NTaxa	7	8	7	6	11
NISP	655	574	305	71	435
Simpson’s Index	0.6921	0.7145	0.6515	0.7875	0.7628

The most common identified species in the assemblage as a whole (Table 1) was the European glass lizard (*Pseudopus apodus*, NISP = 691, 15.9% of the total assemblage) followed by the large whip snake (*Dolichophis jugularis*, NISP = 596, 13.7%), the eastern Montpellier snake (*Malpolon insignitus*, NISP = 371, 8.5%) and to a lesser degree the common viper (*Daboia palaestinae*, NISP = 96, 2%). Taxonomic evenness varied between the EN domestic contexts of the site (Inside and Outside structure II and Loc. 67 area) and the non-domestic Loc. 25 (Simpson’s index of 0.71 for the domestic contexts and 0.79 for Loc. 25, Diversity Permutation Test P < 0.05) (Supplementary Fig. S4). Within the domestic contexts, Loc. 67 area has the lowest evenness values (Simpson’s index of 0.65) and has significantly lower evenness values than Loc. 25 (Diversity Permutation Test P < 0.05). The relative abundance of taxa from these two contexts also differs, with higher abundance of the glass lizard in Loc. 67 (22%) and considerably fewer glass lizards and more colubrids in Loc. 25 (Supplementary Fig. S9). There is also a clear size difference of the centrum length of the vertebrae between the various contexts at the site and the el-Wad Niche sample (one-way ANOVA F = 283 P < 0.05). The significant difference is between the larger vertebrae of the EN domestic contexts and the smaller-sized vertebrae of the non-domestic Loc. 25. The el-Wad Niche assemblage vertebrae are considerably smaller than all other assemblages (Tukey’s Q P < 0.05) (Supplementary Fig. S10).

We now turn to reconstructing the squamate accumulation processes at the site, according to our experimental results. Three of the bone surface modification types that were defined above, types A, C and F, can shed light on the agents of accumulation. Types A and C were both identified as indicating digestion. Type A is very rare at the site (n = 9) but type C is more common (16–33% of the samples; Supplementary Table 7). Digestion abundance and intensity are significantly different in the archaeological vs. pellets datasets (Supplementary Fig. S11). While our Natufian samples yielded 16–34% digested remains, the presumed barn owl pellet remains in the el-Wad Niche yielded 41% digested specimens, and the eagle owl pellets exhibited ca. 60–80% digested specimens (Supplementary Table S7). In addition, low-intensity digestion was significantly overrepresented in all pellets, and severely underrepresented in the three domestic EN samples. Interestingly, the inside context displays abundant signs of moderate and high-intensity digestion. In contrast, the outside, Loc. 67 area and the LN samples show the lowest digestion abundance (24%, 20% and 26% digested bones, respectively) and underrepresentation of all digestion intensities.

Digestion intensity also varied between the common species at the site. The European glass lizard and the large whip snake had low levels of digestion marks, while the eastern Montpellier snake and the common viper both had overrepresentation of moderate and high intensities of digestion. By comparing the digestion intensity of the common species to the actualistic references, a clear distinction was revealed between the European glass lizard and the large whip snake, and the other species (Supplementary Fig. S12).

**Figure 3:**
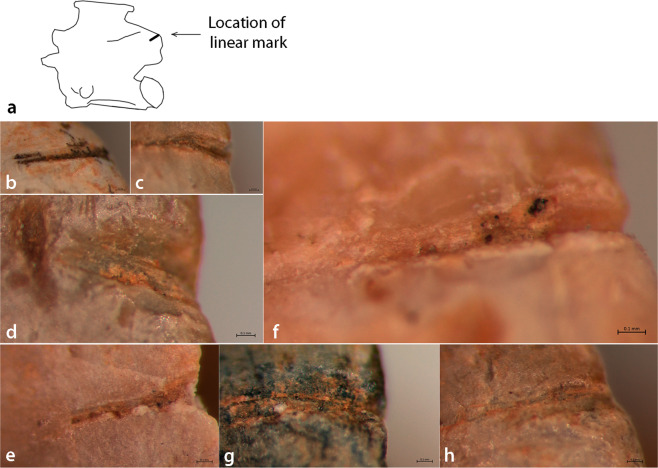
Type F, linear striations on the lateral left postzygapophyses: (**a**) Drawing of location of the mark; (**b–j**) photos of the striations.

Type F, linear marks with a v-shaped cross section on the lateral left postzygapophyses (Fig. 3) were observed on seven non-digested trunk vertebrae originating from outside structure II (n = 5) and Loc. 67 (n = 2) and identified as large whip snake (*Dolichophis Jugularis*, n = 6) and in one case, eastern Montpellier snake (*Malpolon insignitus*, n = 1). The unique linear marks that consistently appeared on the lateral left postzygapophyses were found only in the archaeological material and were not found in the pellets, el-Wad Niche or experimental assemblages (Supplementary Table S7). Linear marks with a v-shaped cross section may result of natural agencies such as trampling^36^, but their relative depth, infrequency and repetitive location on the vertebrae may suggest that they constitute butchery marks, previously unknown for snakes in the Levantine record. This suggestion should be further tested in the future, and, as more squamate archaeofaunas are studied taphonomically, similar modifications may be found.

Following accumulation, the squamate assemblage at the site was exposed to several post-discard agents of modification. Erosion of the bone surfaces is common in both non-digested bones (type B) and digested ones (type C) in all the contexts of the site (Supplementary Table S7). The intensity of erosion slightly varies: the outside context showed higher levels of erosion intensity than all the other contexts (97% of the bones are eroded, 37% of the bones have moderate erosion, 41% great erosion and 8% extreme erosion) while the non-domestic Loc. 25 had slightly lower erosion intensity (90% of the bones are eroded, 42% of the bones have moderate erosion, 21% great erosion and 0.5% extreme erosion), both higher in intensity than any of the experimental assemblages. Erosion was not observed in the fresh pellets and was considerably less common in the el-Wad Niche assemblage (65% of the bones were eroded, 33% were slightly eroded, 20% were moderately eroded and 11% were greatly eroded) (Supplementary Fig. S13).

Cracking (type D) and flaking (type G) were extremely common, but in slightly varying degrees, in all the archaeological samples (>90%). Most of the bones in the el-Wad Niche had slight or no flaking or cracking of the bone surface (Supplementary Figs. S14, S15). The abundance of digestion levels in the assemblage was compared to both cracking and flaking levels and no correlation, or a very weak one, was found (digestion vs. cracking, r = 0.019, p = 0.24; digestion vs. flaking, r = 0.137, p < 0.05) suggesting that there is no clear relationship between digestion intensity and cracking and flaking intensities.

A significant difference was discerned between the breakage patterns of the archaeological material and the actualistic assemblages (the protruding parts breakage index, Kruskal-Wallis’s H = 10.98 P < 0.05). The archaeological material had a considerably higher breakage index (between 0.43–0.67) than both el-Wad Niche (0.31), owl pellets (0.03, 0.09) and the experimental material (between 0.07 and 0.28) presumably due to the observed sediment erosion and trampling processes (Supplementary Fig. S16).

**Figure 4:**
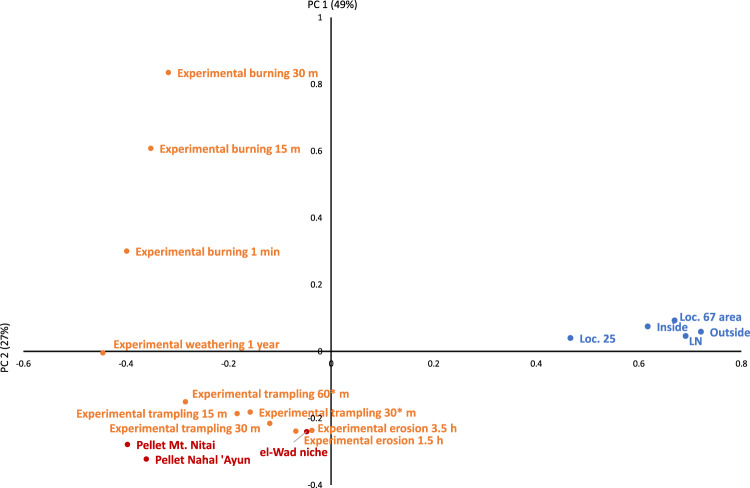
Results of Principal Components Analysis (components 1 and 2) of the different bone modification types in archaeological contexts (blue), pellet assemblages (red) and experimental material (orange).

To obtain a holistic picture of the variance in taphonomic signatures of the archaeological and actualistic samples, we performed a Principal Components Analysis (PCA) of the aforementioned modification types. The first PC, accounting for 49% of variance, shows that the archaeological assemblages clustered together relative to the actualistic ones, and are clearly divergent from them (Fig. 4). The second PC, accounting for 22% of variance, also separates the archaeological samples from the pellet assemblages. The Loc. 25 sample, while clearly separated from the actualistic assemblages, diverges slightly from the other archaeological assemblages suggesting a somewhat different taphonomic history.

**Figure 5:**
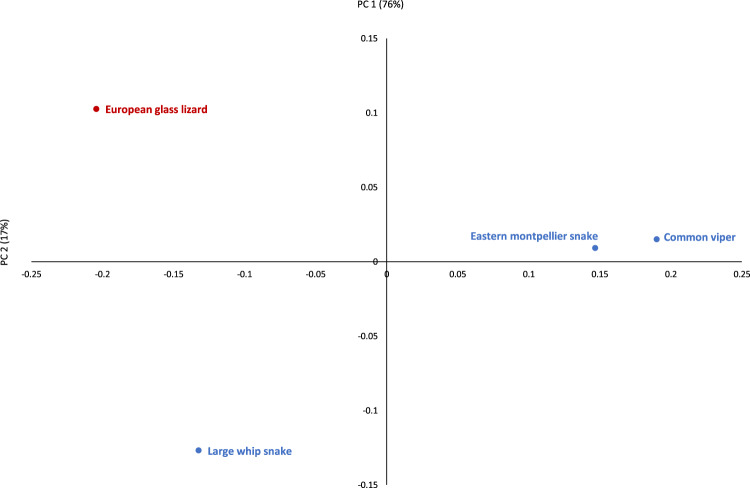
Results of Principal Components Analysis (components 1 and 2) of the different bone modification types in the European glass lizard (red) and the three other common species of the archaeological sample (blue).

The four common species from the entire EWT archaeological assemblage (European glass lizard, large whip snake, eastern Montpellier snake and the common viper) were also plotted (Fig. 5). The first PC, accounting for 76% of variance, separates the European glass lizard and the large whip snake from the other two species, the eastern Montpellier snake and the common viper. The latter two cluster tightly together in PC1 and PC2, suggesting a shared taphonomic history differing from the former species.

## Discussion

The comparison between the experimental material showing the different types of bone surface modifications on squamate vertebrae, and the Natufian archaeological material provides a better understanding of the complex palimpsest of the EWT. Due to the complexity of the overlapping taphonomic processes on the Natufian squamate remains, this comparison is crucial in order to identify and differentiate the taphonomic signatures of accumulation and post-depositional processes.

The experiments enabled us to identify and define several characteristic types of bone surface modifications on the squamate vertebrae, to assign them to accumulation or post-depositional processes and to discern overlapping processes. Specifically, correctly recognizing digestion in fossil material that had subsequently been subjected to various erosional processes is not straightforward. Hence, we designed experiments to compare patterns of post-discard damage (burning, weathering, trampling and erosion) to both untreated and digested bones. By comparing the archaeological assemblage to the actualistic data we showed that the Natufian samples deviated from all actualistic ones. In both the pellets and el-Wad Niche we identified clear taphonomic evidence of digestion (type A). In contrast, in the Natufian assemblage classic digestion marks (type A) are not common. Rather, we found evidence for eroded digestion marks (type C). Even when considering both the “pristine” and “damaged” digestion marks, the evidence for animal predation is still low in the Natufian samples compared to the pellets. The extremely abundant modifications reflecting trampling, erosion and fragmentation (types B, C and E) on squamate vertebrae in the archaeological assemblage indicate that post-discard damage had a major impact on bone taphonomy at the site. This may be related to the high intensity of site occupation (e.g.^37^).

We were also able to identify different intra-site and inter-species patterns in the EWT material. The EN domestic contexts (inside and outside Structure II and Loc. 67 area) are characterized by larger-bodied species, high levels of erosion and breakage, less common occurrence of digestion marks, and by the presence of possible butchery marks (type F). The non-domestic context of Loc. 25 displays a larger number of smaller-bodied species and higher taxonomic evenness, lower levels of erosion and breakage and considerably higher abundance of digestion marks. The locus’ location outside of the Natufian living space close to the overhanging cliff, and its divergence from the domestic contexts suggest that this assemblage was mostly accumulated by avian predation and natural death. This conclusion is congruent with previous studies of other materials in EWT, consistently finding taphonomic and taxonomic differences between Loc. 25 and the domestic contexts. The taphonomic study of micro-mammals showed Loc. 25 to include diverse rodent remains that originated from owl predation, while the domestic contexts included numerous mole-rat (*Spalax* sp.) remains bearing some evidence of human consumption^16^. Similarly, ungulate and small mammal game remains were mostly consumed and discarded in the domestic contexts, with occasional toss of the largest remains forming the Loc. 25 archaeofaunal deposit^37^.

The domestic contexts are more complex; raptor predation can be identified at the site but the differences between the owl pellets, el-Wad Niche and Loc. 25, and the domestic contexts suggest that raptor predation was not the main accumulation agent of all squamate remains in EWT. The abundance of larger animals as indicated by the vertebrae size, and of larger squamate species than usually found in pellets, combined with the possible butchery marks and lower rates of digestion, suggests that some of the remains were accumulated by human consumption.

Taphonomic histories vary also between different species at the site; among the four major species, the sub-venomous eastern Montpellier snake and the venomous common viper have noticeably higher digestion percentages than the European glass lizard and the large whip snake. The high abundance of European glass lizard remains in the EN domestic contexts, most especially Loc. 67 that is associated with human refuse disposal, along with relatively low percentages of digestion marks suggests human consumption. Human consumption of squamate species was previously suggested for the Levantine Pleistocene record^8,13–15,24,38^. However, no clear taphonomic protocol was presented to identify human consumption within a complex palimpsest. Our detailed bone surface modification study along with the contextual taphonomic study conducted for the site of el-Wad provides such protocol, that can be employed in additional assemblages from various sites and accumulation types.

Our experimental results contribute to a better understanding of squamate taphonomy, which we showed is different in key ways from the much better-studied micromammal taphonomy. In small-mammal studies, a high level of digestion is correlated to higher levels of cracking and flaking of the bone surface^35^, but in the case of the experimental and Natufian squamate remains, there is no correlation between the levels of digestion and the levels of cracking and flaking, suggesting that cracking and flaking do not necessarily form as a result of digestion.

This study sheds light on the importance of squamate remains in zooarchaeological studies in the Natufian culture and as part of the archaeological assemblage. This research provides a sound starting point for additional experimental studies that would provide valuable data regarding human consumption of squamate remains (e.g. experimental roasting and butchering), distinction between the predation effects of raptor and mammalian taxa and additional post-depositional bone surface modifications. Further lines of research would integrate multi-taxa analysis and comparison to other micro- and macro-fauna. These will enable us to deepen and refine our understanding of the role of squamates in the zooarchaeological assemblages.

Our study provides important insights into the processes underlying squamate accumulations at EWT, a complex taphonomic system probably akin to many archaeological sites. While some squamates at the site, mostly the eastern Montpellier snake, the viper, and small lizards and snakes probably accumulated by raptor predation and natural death, others were most probably consumed by the inhabitants of the site. The indications for European glass lizard and likely also large whip snake consumption join multiple evidence for other “small game” taxa^16,24,39^ in portraying the remarkable expansion of the diet breadth and subsistence intensification in the terminal Pleistocene Natufian hamlets, on the threshold of agriculture in the Levant.

## Materials and methods

### Materials

The experimental material originated from three different bone accumulations. We obtained fresh European glass lizard (*Pseudopods apodus*) and common viper (*Vipera palaestinae*) carcasses from Tel Assawir (central Israel) and the Hai-Bar nature reserve on Mount Carmel (northern Israel), provided by the Israel Nature and Parks Authority, and subjected them to controlled erosion by sediment, weathering and burning experiments.

Digested squamate remains were retrieved from five eagle owl pellets collected in Nahal ‘Ayun, Israel, and curated in the zoological collections of the Steinhardt Natural History Museum in Tel-Aviv University, and four pellets from Mount Nitai, collected by the Israel Nature and Parks Authority. These constituted remains that have undergone digestion only; we exposed some of the bones in the pellets from Mount Nitai to additional types of damage (erosion by sediment, burning and weathering). In addition, squamate remains were retrieved from the assumed owl micro-vertebrae accumulation el-Wad Niche located in the cliff overhanging the el-Wad Terrace. The niche assemblage represents an intermediate stage between pellets and site accumulation following pellet disintegration^33^. These served as comparative non-anthropogenic accumulation to the archaeological assemblage.

The archaeological squamate remains were collected in the renewed excavations of el-Wad Terrace in 2001–2010^18,40^ and were part of the comprehensive zooarchaeological study conducted at the site^13,37^. The material was retrieved in the University of Haifa excavations, under permission from the Israel Antiquities Authority excavations, licenses G-15/2001, G-8/2002, G-13/2003, G-28/2004, G-13/2005, G-20/2006, G-3/2007, G-2/2008, G-4/2009 and G-5/2010. The spatial and contextual variability in a complex base camp such as el-Wad may affect the taphonomy and taxonomy patterns of the faunal remains^13,16^. Therefore, we sampled the remains from several distinct contexts in the well-preserved Early Natufian (EN) layer. Samples were collected from four stratigraphically similar yet architecturally distinct EN locations at Phases W-6/W-7 of Unit 2^17^: The “inside” and “outside” of Structure II (a small semi-subterranean stone-paved structure), the area of Loc. 67 (a massive stone heap, often cemented by hard, light-gray concretions) and Loc. 25 (a non-domestic heap of stones outside the living compound) (see Fig. 1 for location and^37^ for a detailed description of context selection). To track temporal patterns, a Late Natufian (LN) sample was taken from the overlying archaeological sediments in the same place, Squares O–P/6–7, Unit 1–2 and Phase W-0 in Unit 2 (Fig. 1).

All the squamate remains in this study were retrieved using wet-sieving through 5-mm sieves. Additionally, fine-sieved sediments (1-mm sieve) originating from one square of each relevant context underwent picking in order to have the most complete assemblage and to control size biases.

### Methods

The following analyses employed the Number of Identified Specimens (NISP) as the counting measure, defined as a specimen that can be confidently identified to a skeletal element or part thereof, and can be assigned to species, family or to sub-order (either lizards or snakes). Taxonomic identifications were made using Holman^41^, Szyndlar^42,43^ and the comparative collections of the University of Haifa Laboratory of Archaeozoology, the Tel Aviv University Steinhardt Natural History Museum and the National Natural History Collections at the Hebrew University, Jerusalem.

All archaeological NISP were examined using a stereoscopic microscope (Zeiss Discovery V.8) with a high intensity direct light source, at 10X–80X magnification. Four post-depositional taphonomic processes were experimentally tested: weathering, burning, sediment erosion and trampling. The bones from the experiments were analyzed in the same way and subsequently, we attempted to link the observed patterns to agents of bone deposition and modification, or to the overlap of several agents. To assess body size, the greatest centrum length measurement, following Szyndlar^42^ (Supplementary Fig. S17), was taken in all squamate vertebrae when sufficiently complete.

#### Digestion

Digestion as a bone modifying agent is the result of the predators’ digestive process that modifies the bone through high levels of acidity and digestive enzymes^34^. Due to lack of suitable references, digestion levels were determined according to observations on the squamate bones originating from pellets and scored as low, moderate and high. Low digestion was observed as slight and local perforation of the bone surface with regular edges on the diapophysis and/or the parapophysis. Moderate digestion was observed as extensive deeper perforation of the bone surface with regular edges on the diapophysis and parapophysis and the condyle. High digestion was observed as extensive and deep perforation of the bone surface with regular edges on the diapophysis, parapophysis, condyle and at most cases on the cotyle as well, along with the smoothing of protruding parts of the bone. The deep perforation of the bone surface may cause some breakage of both the diapophysis and the parapophysis (Supplementary Fig. S18).

#### Weathering

Weathering occurs when a bone is exposed to environmental factors such as sun, wind, temperature and humidity that create flaking and splitting of the bone surface^35^. The weathering impact in our experiment was documented according to Andrews’^35^ four stages: stage 0 – no modification, stage 1 – slight splitting of bone parallel to fiber structure; stage 2 – more extensive splitting, little flaking; stage 3 – deep splitting and loss of segments.

We tested the impacts of weathering on two possible accumulation scenarios: bones deposited by natural death at the site (i.e., unmodified carcasses subjected to weathering) and bones deposited by eagle owl predation (i.e., bones that underwent digestion before weathering). The former scenario was tested on a fresh, fleshed and unmodified European glass lizard (*Pseudopus apodus*) carcass. The latter was tested on 15 digested coin-marked snake (*Hemorrhois nummifer*) vertebrae retrieved from a modern eagle owl pellet, which exhibited signs of digestion prior to the experiment (digestion marks and polish).

We kept an untreated control sample of the European glass lizard carcass segment and some digested coin-marked snake vertebrae and subjected the rest to subaerial weathering in as similar environment as possible to our archaeological case study. The specimens were placed in a protective net (that prevented movement but still allowed exposure to the elements) at the el-Wad site, ca. 5 m north-west of our excavation, and were monitored every month for 12 months. We recorded carcass completeness as following: 5 – the carcass was in the same condition as when it was placed in the beginning of the experiment; 4 – scales were deteriorated or completely removed; 3 – scales were no longer articulated but skeletal remains were articulated; 2 – the skeleton was mostly articulated; 1 – bones were either non-articulated or mostly non-articulated. During every inspection we recorded the effects of weathering on bone based on the aforementioned Andrews scale (Supplementary Table S1).

#### Burning

Bone charring and calcination may result from various causes: incidental exposure to fire due to association with a fireplace or a hearth, roasting an animal as part of food preparation or discarding food debris into combustion features^16,44^. In our case study, most of the burnt non-squamate remains (identified according to bone color) were shown to have been unintentionally impacted by combustion activities^37^. Significantly, burning may produce deep cracks on the bone surface, as previously suggested for weathering and digestion^34^, so it is important to differentiate taphonomically between the three.

The process of burning was tested to check several factors:

1. Are cracks resulting from heating similar to those resulting from raptor digestion and weathering?
2. What combination of temperature and time are needed to produce brown, brown-black, black, gray and white coloring on squamate vertebrae?
3. Do non-digested and digested vertebrae react similarly to these time/temperature combinations?

To characterize burning effects, following Shahack-Gross *et al*.^32^, water-cleaned bones from a fresh common viper along with digested coin-marked snake bones were placed in a scientific muffle furnace (FX-14 Daihan) for varying temperatures and times (Supplementary Table S2).

#### Erosion by sediment

Erosion by sediment was tested on each of the following: fresh common viper (*Vipera palaestinae*) disarticulated bones, digested bones, and burnt bones from the previous experiment. All the samples were placed in a plastic container attached to an Intelli-mixer (RM-2L) with dark brown silty clay loam sediment with small gravel originating from the site and were rotated for either 1.5 or 3.5 hours. Bone-completeness (1–4, when 4 is a complete bone) and breakage patterns (complete break of a specific part of the bone, rounding of the bone or erosion of the outer layer of the bone) were documented.

#### Trampling

Trampling was tested by placing natural, digested and burnt bones on sediment originating from the site and stepped on repeatedly by a single person for 15, 30 and 60 minutes. All the sediment was later sieved to retrieve all bone parts. Breakage patterns were documented using both bone-completeness index (1–4, with 4 a complete bone) and protruding-parts breakage index. The protruding parts of the vertebrae body are the neural spine, prezygapophyseal articular facet (right and left sides), prezygapophysis (right and left sides) and postzygapophysis (right and left sides) and these are more prone to breakage. Each part is 1 in the index (7 = all the previously mentioned parts are broken). The breakage index was calculated according to the level of bone completeness (following^45^): the number of complete bones (protruding parts index = 0) is multiplied by 0, the number of bones with protruding parts index 1 is multiplied by 1, the number of bones with protruding parts index 2 is multiplied by 2, etc. The resulting number is divided by the total sum of calculated bones multiplied by 7.

#### Statistical tests

Taxonomic evenness values in the different contexts of the site (domestic and non-domestic) was examined using Simpson’s Index and the diversity permutation test was used to test species evenness between the non-domestic loc. 25 to each of the domestic contexts. We employed principal component analysis (PCA) to compare all the bone surface modification indices (digestion, burning, erosion, cracking, flaking, and completeness) for different samples. The indices for the PCA were calculated in the same manner as the aforementioned breakage index. The statistical analyses were conducted using PAST software^46^.

## Supplementary information

Supplementary information.

## References

[1] Biton R, Bailon S, Goren-Inbar N, Sharon G, Rabinovich R (2019). Pleistocene amphibians and squamates from the Upper Jordan Rift Valley, Gesher Benot Ya’aqov and Nahal Mahanayeem Outlet (MIS 20–18 and MIS 4/3). Quaternary Res..

[2] Blain HA, Bailon S, Agusti J (2007). Anurans and squamate reptiles from the latest early Pleistocene of Almenara-Casablanca-3 (Castellón, East of Spain). Systematic, climatic and environmental considerations. Geodiversitas.

[3] Blain HA, Bailon S, Cuenca-Bescós G (2008). The Early–Middle Pleistocene palaeoenvironmental change based on the squamate reptile and amphibian proxies at the Gran Dolina site, Atapuerca, Spain. Palaeogeo. Palaeoclimat. Palaeoeco..

[4] Blain HA (2009). Long-term climate record inferred from early-middle Pleistocene amphibian and squamate reptile assemblages at the Gran Dolina Cave, Atapuerca, Spain. J. Hum. Evol..

[5] Blain HA (2010). Climate and environment of the earliest West European hominins inferred from amphibian and squamate reptile assemblages: Sima del Elefante Lower Red Unit, Atapuerca, Spain. Quaternary Sci. Rev..

[6] Blain HA (2013). Climatic conditions for the last Neanderthals: Herpetofaunal record of Gorham’s Cave, Gibraltar. J. Hum. Evol..

[7] Maul L (2015). Palaeoecological and biostratigraphical implications of the microvertebrates of Qesem Cave in Israel. Quaternary Int..

[8] Monchot H, Bailon S, Schiettecatte J (2014). Archaeozoological evidence for traditional consumption of spiny-tailed lizard (Uromastyx aegyptia) in Saudi Arabia. J. Archaeol. Sci..

[9] Smith KT, Maul LC, Barkai R, Gopher A (2013). 2013. To catch a chameleon, or actualism vs. natural history in the taphonomy of the microvertebrate fraction at Qesem Cave, Israel. J. Archaeol. Sci..

[10] Stoetzel E, Denys C, Bailon S, El Hajraoui MA, Nespoulet R (2012). Taphonomic analysis of amphibian and squamate remains from El Harhoura 2 (Rabat‐Témara, Morocco): Contributions to palaeoecological and archaeological interpretations. Int. J. Osteoarchaeol..

[11] Bailon S, Aouraghe H (2002). Amphibiens, chéloniens et squamates du Pléistocène Supérieur d’El Harhoura I (Témara, Maroc). Geodiversitas.

[12] Bailon, S. La faune de vertébrés du Pléistocène moyen de la Grotte des Rhinocéros, Casablanca, Maroc. 1 - les Squamates (Reptilia). In Préhistoire de Casablanca. 1- La Grotte des Rhinocéros (fouilles 1991 et 1996). Villes et Sites Archéologiques du Maroc (eds. Raynal, J. P., Mohib, A.) vol. I, 87 (2016).

[13] Yeshurun R, Bar-Oz G, Weinstein-Evron M (2014). Intensification and sedentism in the terminal Pleistocene Natufian sequence of el-Wad Terrace (Israel). J. Hum. Evol..

[14] Tchernov, E. Commensal animals and human sedentism in the Middle East. In Animals and Archaeology 3: Early Herders and their Flocks (eds. Clutton-Brock J. & Grigson C.) 91–115 (BAR International Series 202, British Archaeological Society, Oxford (1984).

[15] Stiner, M. C. The Faunas of Hayonim Cave, Israel: A 200,000-Year Record of Paleolithic Diet, Demography, and Society (No. 48) (Harvard University Press (2005).

[16] Weissbrod L, Bar-Oz G, Yeshurun R, Weinstein-Evron M (2012). Beyond fast and slow: The mole rat Spalax ehrenbergi (order Rodentia) as a test case for subsistence intensification of complex Natufian foragers in southwest Asia. Quatern. Int..

[17] Garrod DAE (1957). The Natufian culture: the life and economy of a Mesolithic people in the Near East. P. Brit. Acad..

[18] Weinstein-Evron M (2018). After 80 years – deeper in the Natufian layers of el-Wad Terrace, Mount Carmel, Israel. J. Isr. Prehist. Soc..

[19] Flannery, K. V. Origins and ecological effects of early domestication in Iran and the Near East. In The Domestication and Exploitation of Plants and Animals (eds. Ucko, P. J. & Dimbleby, G. W.) 73–100 (Duckworth, London (1969).

[20] Bar-Oz, G. Epipaleolithic Subsistence Strategies in the Levant: A Zooarchaeological Perspective (Brill Academic Pub (2004).

[21] Davis, S. J. M., Lernau, O. & Pichon, J. The animal remains: new light on the origin of animal husbandry. In Le Gisement de Hatoula en Judée Occidentale, Israël (eds. Lechevalier, M. & Ronen, A.) 83–100 (Association Paléorient, Paris (1994).

[22] Munro ND (2004). Zooarchaeological measures of hunting pressure and occupation intensity in the Natufian: implications for agricultural origins. Curr. Anthropol..

[23] Stiner MC, Munro ND, Surovell TA, Tchernov E, Bar-Yosef O (1999). Paleolithic population growth pulses evidenced by small animal exploitation. Science.

[24] Stiner MC, Munro ND, Surovell TA (2000). The Tortoise and the hare: Small-game Use, the Broad-Spectrum Revolution, and Paleolithic demography. Curr. Anthropol..

[25] Bar‐Yosef O (1998). The Natufian culture in the Levant, threshold to the origins of agriculture. Evol. Anthropol..

[26] Biton, R. & Bailon, S. Frogs, lizards and snakes from Eynan - background fauna or more? ASWA conference, Barcelona (2019).

[27] Pinto-Llona AC, Andrews PJ (1999). Amphibian taphonomy and its application to the fossil record of Dolina (middle Pleistocene, Atapuerca, Spain). Palaeogeo. Palaeoclimat. Palaeoeco..

[28] Castillo C, Martın-González E, Coello JJ (2001). Small vertebrate taphonomy of La Cueva del Llano, a volcanic cave on Fuerteventura (Canary Islands, Spain). Palaeoecological implications. Palaeogeo. Palaeoclimat. Palaeoeco..

[29] Yeshurun, R., Bar-Oz, G., Kaufman, D. & Weinstein-Evron, M. Domestic refuse maintenance in the Natufian: faunal evidence from el-Wad Terrace, Mount Carmel In Natufian Foragers in the Levant: Terminal Pleistocene Social Changes in Western Asia 118–138 (eds. Bar-Yosef, O. & Valla, F. R.) (International Monographs in Prehistory, Ann Arbor (2013).

[30] Zohar I, Ovadia A, Goren-Inbar N (2016). The cooked and the raw: A taphonomic study of cooked and burned fish. J. Archaeo. Sci.: Reports.

[31] Reidsma FH, van Hoesel A, van Os BJ, Megens L, Braadbaart F (2016). Charred bone: Physical and chemical changes during laboratory simulated heating under reducing conditions and its relevance for the study of fire use in archaeology. J. Archaeo. Sci.: Reports.

[32] Shahack-Gross R, Bar-Yosef O, Weiner S (1997). Black-coloured bones in Hayonim Cave, Israel: differentiating between burning and oxide staining. J. Archaeol. Sci..

[33] Weissbrod L, Dayan T, Kaufman D, Weinstein-Evron M (2005). Micromammal taphonomy of el-Wad Terrace, Mount Carmel, Israel: distinguishing cultural from natural depositional agents in the Late Natufian. J. Archaeol. Sci..

[34] Fernandez-Jalvo, Y. & Andrews, P. Atlas of Taphonomic Identifications: 1001+ Images of Fossil and Recent Mammal Bone Modification (Springer (2016).

[35] Andrews, P. O *Caves and Fossils* (London: Natural History Museum Publications (1990).

[36] Domínguez-Rodrigo M, De Juana S, Galan AB, Rodríguez M (2009). A new protocol to differentiate trampling marks from butchery cut marks. J. Archaeol. Sci..

[37] Yeshurun R, Bar-Oz G, Kaufman D, Weinstein-Evron M (2014). Purpose, permanence, and perception of 14,000-year-old architecture. Curr. Anthropol..

[38] Speth J (2012). Middle Palaeolithic subsistence in the Near East: zooarchaeological perspectives–past, present and future. Before Farming.

[39] Yeshurun R, Bar-Oz G, Weinstein-Evron M (2009). The role of foxes in the Natufian economy: a view from Mount Carmel, Israel. Before. Farming.

[40] Weinstein-Evron M (2007). After 70 years: new excavations at the el-Wad Terrace, Mount Carmel, Israel. J. Isr. Prehist. Soc..

[41] Holman, J. A. Pleistocene Amphibians and Reptiles in Britain and Europe (Vol. 38) (Oxford University Press (1998).

[42] Szyndlar Z (1984). Fossil snakes from Poland. Acta Zoologica Cracoviensia.

[43] Szyndlar Z (1991). A review of Neogene and Quaternary snakes of central and eastern Europe. Part 1: Scolecophidia, Boidae, Colubrinae. Estud. Geol..

[44] Stiner MC, Kuhn SL, Weiner S, Bar-Yosef O (1995). Differential burning, recrystallization, and fragmentation of archaeological bone. J. Archaeol. Sci..

[45] Costamagno, S., Théry-Parisot, I., Brugal, J. P. & Guibert, R. Taphonomic consequences of the use of bones as fuel. Experimental data and archaeological applications. In Biosphere to lithosphere: new studies in vertebrate taphonomy (ed. O’Connor, T.) 51–62 (Oxbow Books Oxford (2005).

[46] Hammer Ø, Harper DA, Ryan PD (2001). PAST: Paleontological statistics software package for education and data analysis. Palaeontol. Electron..

