# Supplementary material for: Squamate bone taphonomy: A new experimental framework and its application to the Natufian zooarchaeological record
Source: Sci Rep. 2020 Jun 10;10:9373. doi: 10.1038/s41598-020-66301-5 (PMC7287132; doi:10.1038/s41598-020-66301-5)

Squamate bone taphonomy: A new experimental framework and its application to the Natufian zooarchaeological record

Ma'ayan Lev*, Mina Weinstein-Evron and Reuven Yeshurun

Supplementary materials

Table S1. Observations on the glass lizard carcass weathering experiment (start date: 22.11.2018).

Date of observations	Carcass completeness	Cracking intensity
22.11.2018	5	no marks on the bone surface
6.12.2018	5	no marks on the bone surface
24.12.2019	5	no marks on the bone surface
17.1.2019	4	no marks on the bone surface
29.1.2019	4	no marks on the bone surface
1.2.2019	4	no marks on the bone surface
19.2.2019	3	no marks on the bone surface
28.2.2019	3	no marks on the bone surface
2.5.2019	2	no marks on the bone surface
25.6.2019	2	no marks on the bone surface
12.8.2019	1	very slight cracks
1.12.2019	1	slight cracks

Note: Carcass completeness stages according to 5- the carcass is in the condition it was placed in the beginning of the experiment, 4- scales are deteriorated or completely removed, 3- scales no longer articulated but skeleton remains articulated, 2- the skeleton is mostly articulated, 1- bones are either non-articulated or mostly non-articulated.

Table S2. Data for burning experiment, showing the sample's origin, burning time, burning temperature and resulting bone modifications.

Catalog number	Nature of the bone	Time (minutes)	Temperature (^{0}C)	Color	Cracking intensity
1	untreated	1	350	Brown-black	Moderate
2	untreated	1	350	Brown-black	Moderate
3	untreated	1	300	Brown	Slight
4	untreated	1	300	Brown	Slight
5	untreated	1	400	Black	Moderate
6	untreated	1	400	Black	Moderate
7	untreated	1	550	Black-gray	Great
8	untreated	1	550	Black-gray	Great
9	untreated	15	550	Gray	Great
10	untreated	15	550	Gray	Great
11	untreated	30	800	White	Extreme
12	untreated	30	800	White	Extreme
13	digested	1	300	Brown	None
14	digested	1	300	Brown	Slight
15	digested	1	350	Brown-black	Slight
16	digested	1	350	Brown-black	Slight
17	digested	1	400	Black	Moderate
18	digested	1	400	Black	Moderate
19	digested	15	550	Gray	Great
20	digested	15	550	Gray	Great
21	digested	30	800	White	Extreme
22	digested	30	800	White	Extreme

Table S3. Data from erosion by sediment experiment.

Catalog number	Nature of bone	Time (hours)	Erosion intensity
1	Untreated	1.5	Moderate
2	Untreated	1.5	Moderate
3	Digested	1.5	Moderate
4	Burnt	1.5	Great
5	Burnt	1.5	Great
6	Burnt	1.5	Extreme
7	Untreated	1.5	Moderate
8	Untreated	1.5	Moderate
9	Burnt	1.5	Great
10	Burnt	1.5	Extreme
11	Digested	1.5	Great
12	Digested	1.5	Great
13	Untreated	3.5	Great
14	Untreated	3.5	Great
15	Burnt	3.5	Extreme
16	Burnt	3.5	Great
17	Digested	3.5	None
18	Digested	3.5	None
19	Untreated	3.5	Great
20	Digested	3.5	Great

Table S4. Data for trampling experiment.

Catalog number	Nature of bone	Time (minutes)	Erosion intensity	Breakage index
1	Untreated	15	Moderate	0
2	Untreated	15	None	0
3	Untreated	15	Moderate	1
4	Untreated	15	Moderate	1
5	Burnt	15	Great	5
6	Burnt	15	Moderate	0
7	Burnt	15	Moderate	1
8	Burnt	15	Great	2
9	Untreated	15	Moderate	0
10	Untreated	15	Slight	0
11	Burnt	15	Moderate	4
12	Burnt	15	Moderate	4
13	Digested	15	None	0
14	Digested	15	None	0
15	Untreated	30	moderate	1
16	Untreated	30	Slight	0
17	Burnt	30	Great	1
18	Burnt	30	Extreme	4
19	Digested	30	Slight	0
20	Digested	30	Great	0
21	Untreated	30	Slight	0
22	Untreated	30	Great	0
23	Burnt	30	Extreme	5
24	Burnt	30	Great	4
25	Burnt	30	Moderate	7
26	Digested	30	None	0
27	Digested	30	None	0
28*	Digested	30	Slight	2
29*	Digested	30	Moderate	0
30*	Digested	30	Slight	1
31*	Digested	30	Slight	0
32*	Digested	30	Slight	0
33*	Digested	30	None	0
34*	Digested	30	Slight	1
35*	Digested	30	None	3
36*	Untreated	30	Moderate	4
37*	Untreated	30	Moderate	0
38*	Untreated	30	Extreme	1
39*	Burnt	30	Slight	7
40*	Burnt	30	Slight	7
41*	Digested	60	Slight	0
42*	Digested	60	Slight	0
43*	Digested	60	None	0
44*	Digested	60	None	0
45*	Digested	60	Slight	1
46*	Digested	60	Slight	0
47*	Digested	60	Slight	0

48*	Digested	60	Slight	1
49*	Untreated	60	Moderate	0
50*	Untreated	60	Moderate	0
51*	Untreated	60	Moderate	0
52*	Burnt	60	Extreme	6
53*	Burnt	60	Bone not retrieved	
54*	Burnt	60	Bone not retrieved	
55*	Burnt	60	Bone not retrieved	

Table S5. Digestion marks intensities in Mount Nitai and Nahal ‘Ayun pellets (number of identified specimens).

	pellet Mt. Nitai	Pellet Nahal ‘Ayun, eastern slope	Pellet Nahal ‘Ayun eastern slope below the cliff	Total	%
No digestion	14	7	8	29	19%
Low digestion	49	20	18	87	57%
Moderate digestion	10	8	16	34	22%
High digestion	0	1	1	2	1%

Table S6. Data for el-Wad niche assemblage (number of identified specimens).

	Erosion intensity	Digestion intensity	Protruding parts breakage index
None	95	138	71
Slight	89	89	101
Moderate	53	33	50
Great	29	13	28
Extreme	2	0	17

Table S7. Bone surface modification for the el-Wad Terrace contexts, el-Wad niche, Mt. Nitai and Nahal ‘Ayun pellets and experimental material (number of identified specimens).

	Inside	Outside	Loc. 67 area	Loc.25	LN	el-Wad niche	pellet Mt. Nitai	pellet Nahal 'Ayun	Experimental erosion 1.5 h	Experimental erosion 3.5 h	Experimental trampling 15 min	Experimental trampling 30 min	Experimental trampling 30* min	Experimental trampling 60* min	Experimental weathering	Experimental burning
NISP (vert only)	1391	1026	596	207	690	269	73	79	12	8	14	13	13	15	10	22
Type A	6	1	1	1	0	19	50	64	0	0	0	0	0	0	0	0
%	0.44 %	0.10%	0.17 %	0.48 %	0.00 %	7.06 %	68.49 %	81.01 %	0%	0%	0%	0%	0%	0%	0%	0%
Slight	0	0	0	0	0	17	40	38								
Moderate	3	1	1	1	0	2	10	24								
Great	3	0	0	0	0	0	0	2								
Type B	759	702	412	127	512	61	0	0	9	5	11	7	5	4	0	0
%	54.6 %	68.4%	69.1 %	61.4 %	74.2 %	22.7 %	0%	0%	75%	63%	79%	54%	38%	27%	0%	0%
Slight	165	101	114	47	117	28			0	0	1	2	2	0		
Moderate	306	259	155	55	174	20			4	0	8	2	2	3		
Great	261	283	119	24	196	13			3	4	2	3	0	0		
Extreme	27	59	24	1	25	0			2	1	0	2	1	1		
Type C	465	246	120	53	108	112	0	0	3	1	0	2	6	6	0	0
%	33%	24%	20%	26%	16%	42%	0%	0%	25%	13%	0%	15%	46%	40%	0%	0%
Slight	69	6	8	6	4	61			0	0	0	1	5	6		
Moderate	242	104	67	30	65	33			1	0	0	0	1	0		
Great	139	117	41	17	36	16			2	1	0	1	0	0		
Extreme	15	19	4	0	3	2			0	0	0	0	0	0		
Type D	1349	1004	573	198	653	91	0	0	0	0	0	0	0	0	3	22

%	97%	98%	96%	96%	95%	34%	0%	0%	0%	0%	0%	0%	0%	0%	30%	100%
None	21	13	15	8	34	176									7	1
Slight	617	565	376	130	437	90									3	5
Moderate	480	261	132	50	150	1									0	6
Great	184	141	59	13	55	0									0	6
Extreme	68	37	6	5	11	0									0	4
Type F	0	5	2	0	0	0	0	0	0	0	0	0	0	0	0	0
%	0.0%	0.5%	0.3%	0.0%	0.0%	0%	0%	0%	0%	0%	0%	0%	0%	0%	0%	0%
Type G	1300	995	541	199	656	122	0	0	0	0	0	0	0	0	0	0
%	93.5 %	0.969786	90.8 %	96.1 %	95.1 %	45.4 %	0%	0%	0%	0%	0%	0%	0%	0%	0%	0%
None	83	20	48	7	29	146										
Slight	452	349	254	67	295	114										
Moderate	618	458	215	96	291	6										
Great	195	167	67	33	65	2										
Extreme	35	21	5	3	5	0										
burning																
n	510	479	429	57	380	0	0	0								
%	37%	47%	72%	28%	55%	0%	0%	0%								
complete	11	2	3	1	0	71	62	54	7	5	7	7	5	12	10	22
near complete	311	322	322	96	152	137	11	23	3	1	4	2	5	2	0	0
broken	152	264	170	108	162	35	0	2	0	0	3	3	2	0	0	0
very broken	70	228	20	17	196	25	0	0	2	2	0	0	2	1	0	0
of completeness	544	738	516	177	510	268	72	79	12	8	14	12	14	15	10	22
1	114	42	60	15	81	4	0	0	1	2	0	1	2	0	0	0
2	364	318	154	62	223	36	0	0	0	0	0	0	0	1	0	0

3	627	552	281	76	320	58	0	0	3	1	4	2	2	0	0	0
4	280	108	97	54	552	171	72	79	8	5	10	10	9	11	10	22
digestion																
light	36	18	3	22	27	85	40	38								
Moderate	176	95	49	12	45	33	10	24								
high	263	142	70	22	38	13	0	2								
total	475	255	122	56	110	131	50	64								
%	34%	25%	20%	27%	16%	49%	68%	81%								

Table S8. Taxonomic composition of the el-Wad Terrace squamate sample of baskets that underwent both 5mm and 1mm collection (in NISP).

Species	Inside	Outside	L.67 area	L.25	LN
Lizard					
European glass lizard (*Pseudopus apodus*)	30	53	133	11	81
Roughtail rock agama (*Stellagama stellio ssp.*)	9	18	13	8	30
Schneider's skink (*Eumeces schneideri pavimentatus*)		2			4
Levant green lizard (*Lacerta media israelica*)	1		1		
Common chameleon (*Chamaeleo chamaeleon recticrista*)		1			1
Scincidae	1				2
Lacertidae		1		1	
Other lizards	12	6	9	5	68
Snake					
Large whip snake (*Dolichophis jugularis*)	17	41	79	15	90
Eastern Montpellier snake (*Malpolon insignitus*)	24	39	39	17	68
Coin-marked snake (*Hemorrhois nummifer*)		3			
Common viper (*Daboia palaestinae*)	4	8	7	2	31
Javelin sand boa (*Eryx jaculus*)				6	
Dice snake (*Natrix tessellata*)					1
Colubridae-Colubrinae	13	11	20	5	39
Colubridae- Psamophiininae		2	1	2	1
Colubridae-Elaphe			1		
Colubridae	16	44	57	27	145
Other snakes	142	211	255	93	496
Total number of NISP	269	440	615	192	1022
Species only					
NTaxa	5	8	6	6	8
NISP	84	165	272	59	306
Simpson's Index	0.7361	0.7644	0.653	0.7877	0.774

Figure S1. Weathering experiment. Carcass completeness stages. (a) Carcass completeness level 5; (b) Carcass completeness level 4; (c) Carcass completeness level 3; (d) Carcass completeness level 2; (e) Carcass completeness level 1.

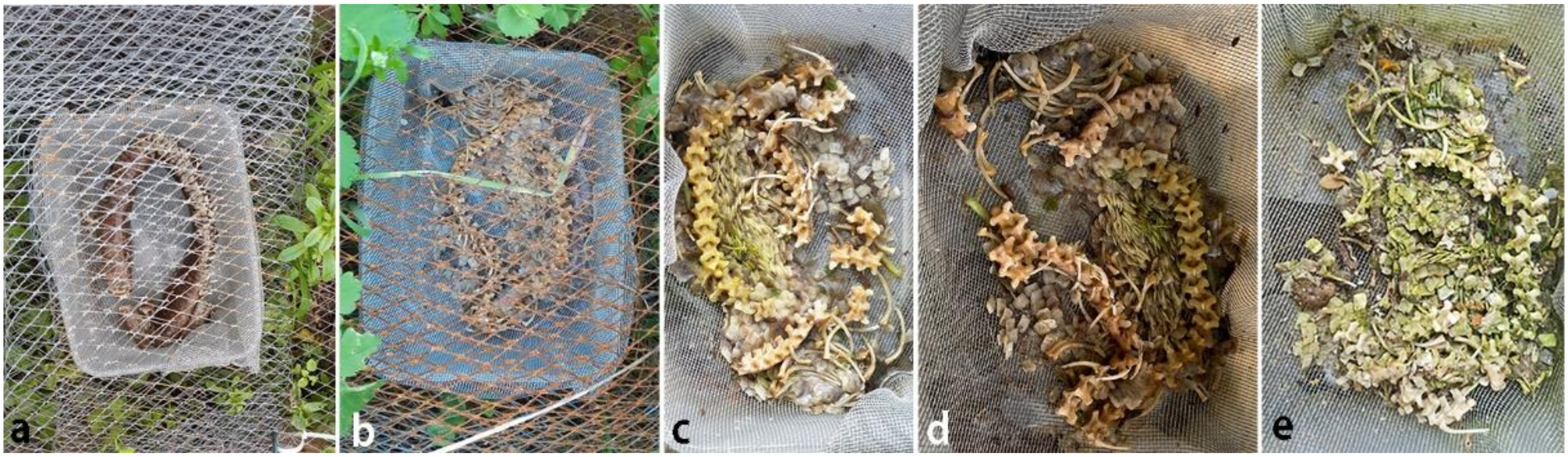

Figure S2. Experimental burning of digested and non-digested snake trunk vertebrae: a–e, Cranial view of non-digested bones; f–j, Cranial view of digested bones. First row 10x magnification, second row 50x magnification.

Temperature/time combinations: a,f- 300°/1 min, b,g- 350°/1 min, c,h- 400°/1 min, d,i- 550°/15 min, e,j- 800°/30 min.

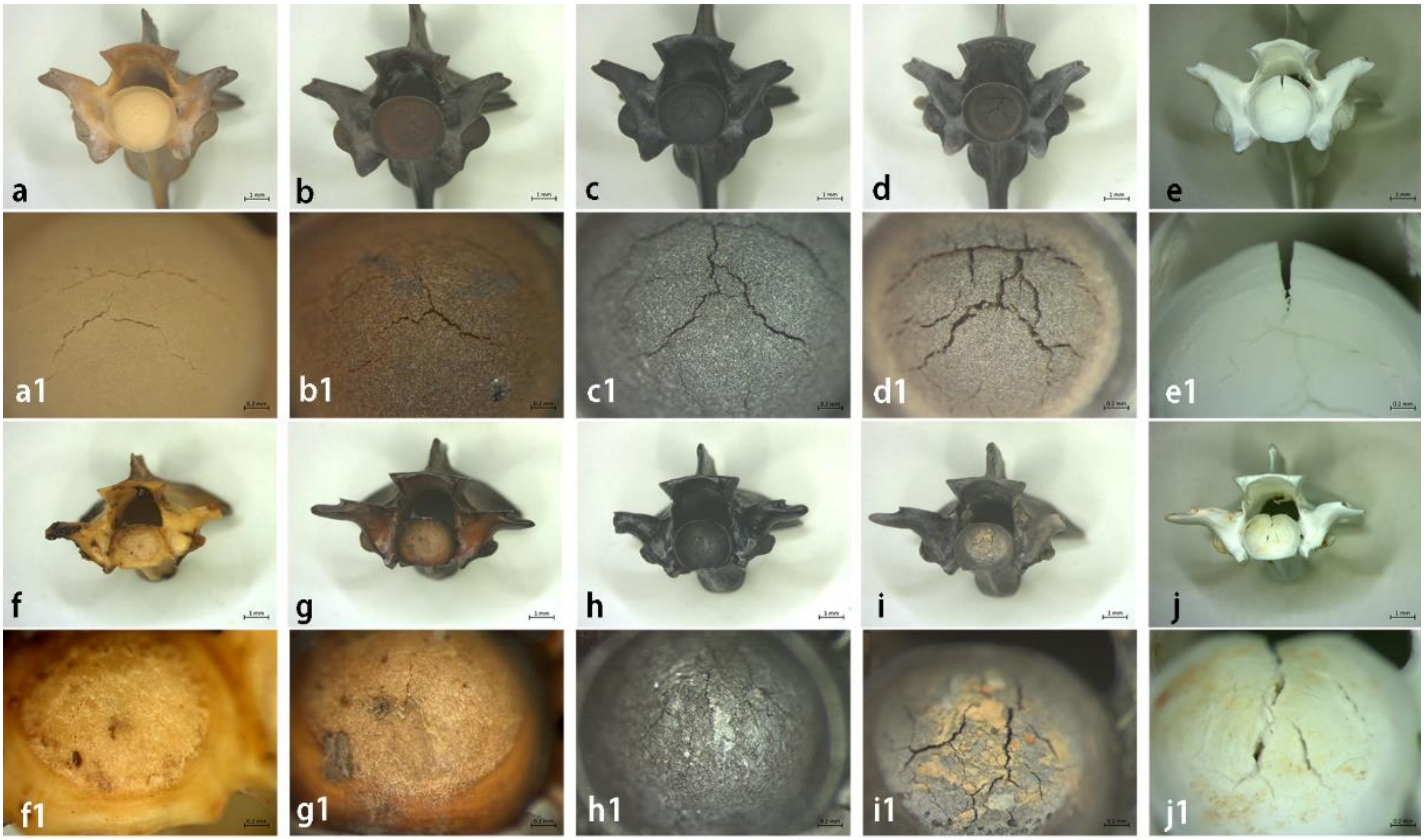

Figure S3. The relationship between burning intensity (conculcated by the color of the bone, untreated=0, brown=1, brown-black=2, black=3, black-gray=4, gray=5 and white=6) and cracking intensity for each of the specimens in the burning experiment (data from Supplementary Table S2).

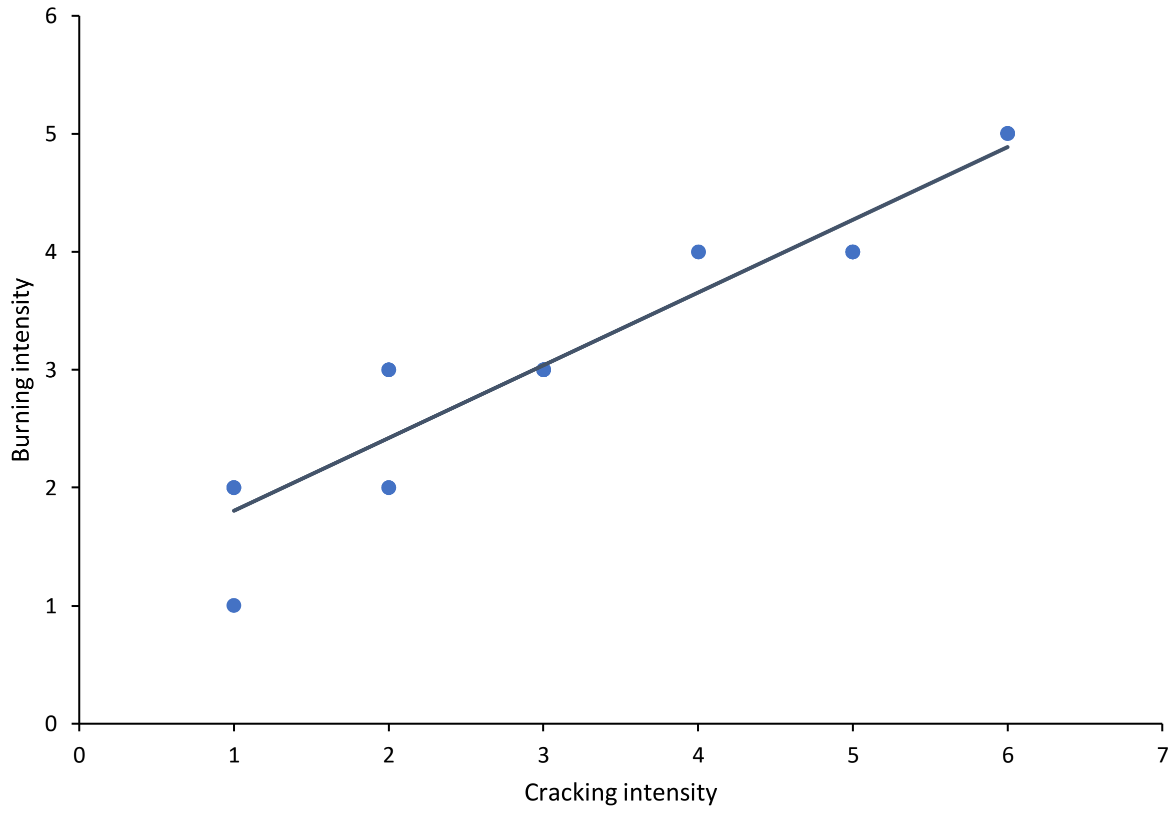

Figure S4. Erosion by sediment experiment. Degrees of erosion intensity vs. The initial nature of the bone: (a) Slight erosion; (b) Moderate erosion; (c) Great erosion; (d) Extreme erosion on untreated bones (row 1), and digested bones (row 2).

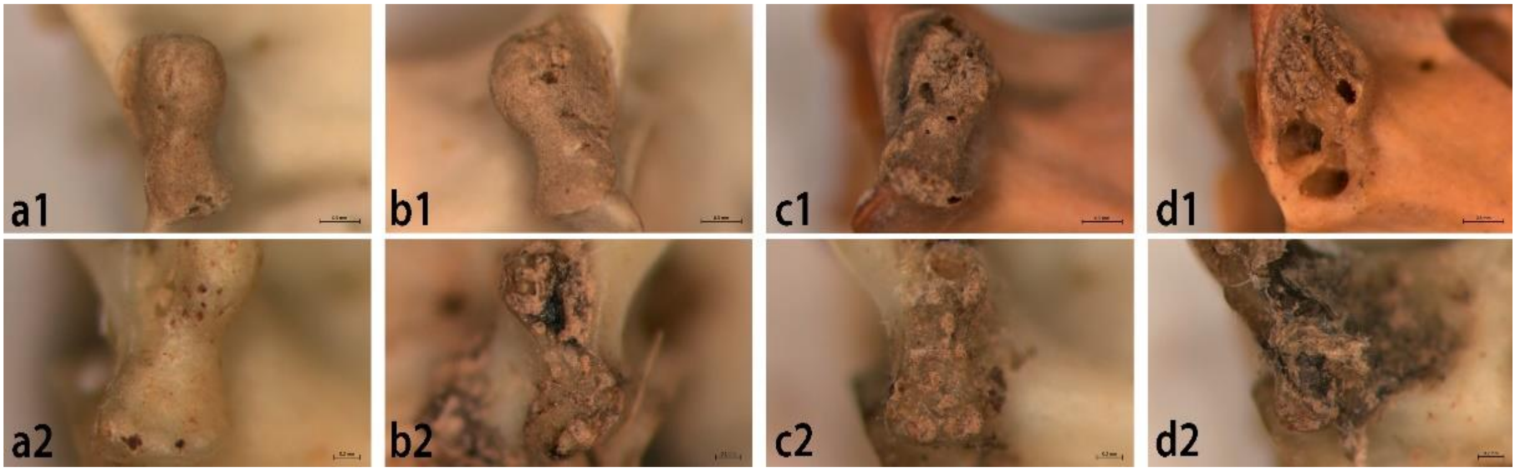

Figure S5. Trampling experiment. Degrees of erosion intensity (slight to extreme): (a) Slight erosion; (b) Moderate erosion; (c) Great erosion; (d) Extreme erosion on untreated bones (row 1), and digested bones (row 2).

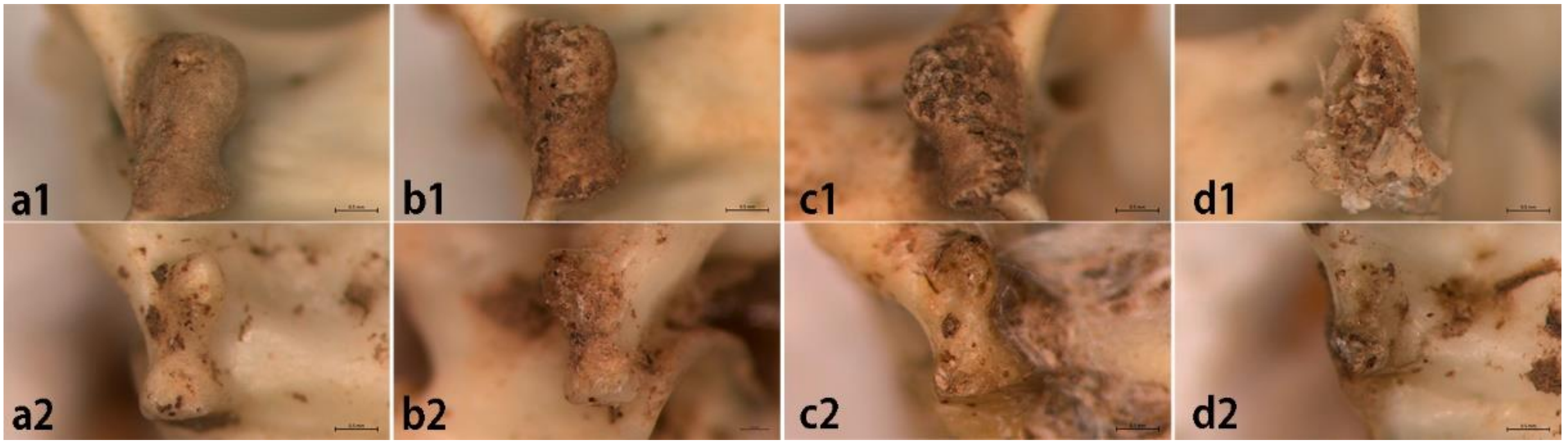

Figure S6. Breakage index for untreated, digested and burnt bones from the trampling experiment. Data from supplementary table S4.

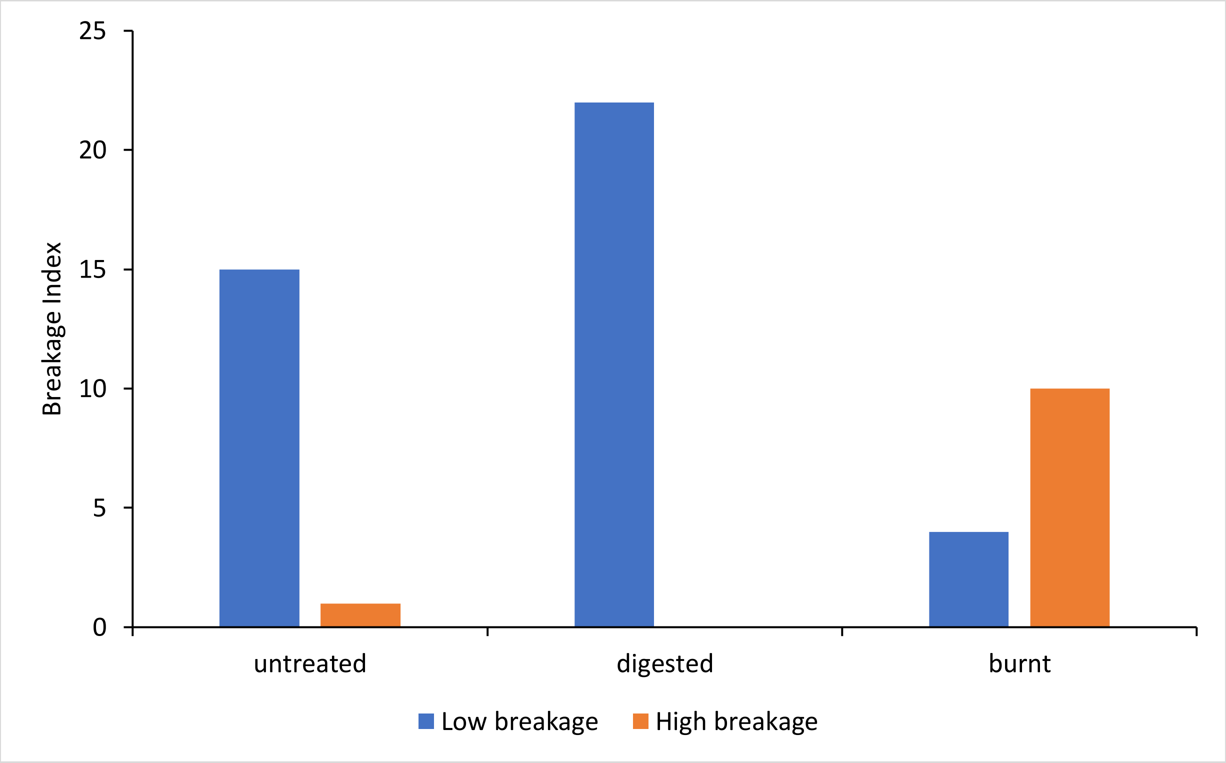

Figure S7. Digestion by eagle owl: Perforations with regular edges on the bone surfaces: (a) Diapophysis and parapophysis (b) Condyle (c) Cotyle.

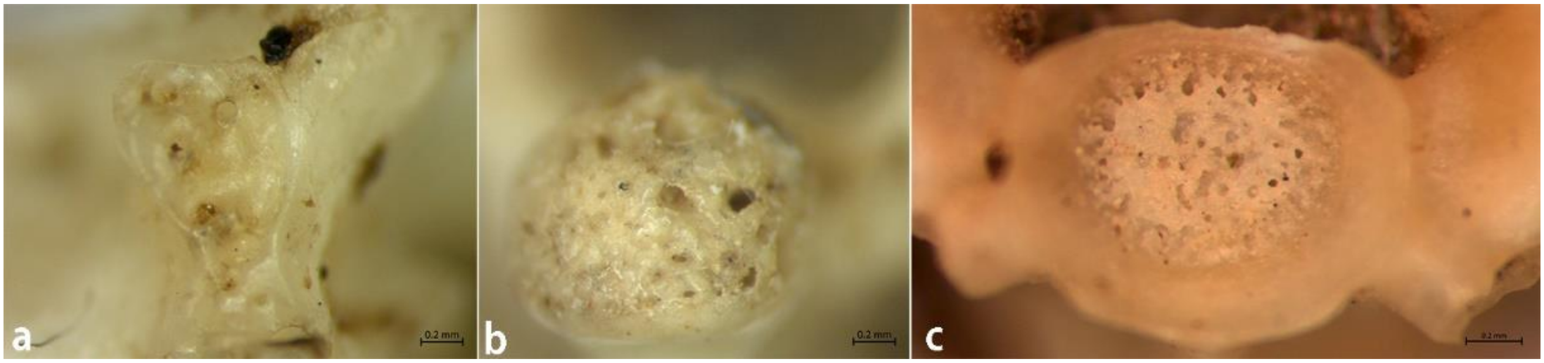

Figure S8. Species evenness (Simpson's index) for the EN domestic contexts (Inside and outside structure II and Loc. 67 area) and the non-domestic Loc.25, data from Table S8.

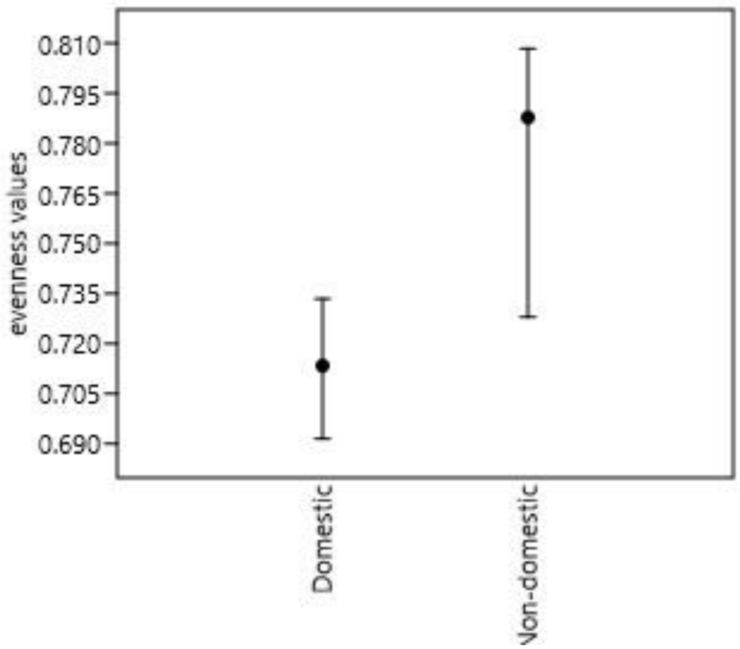

Figure S9. Relative abundance of taxa in the different contexts of the site, Data from table 1.

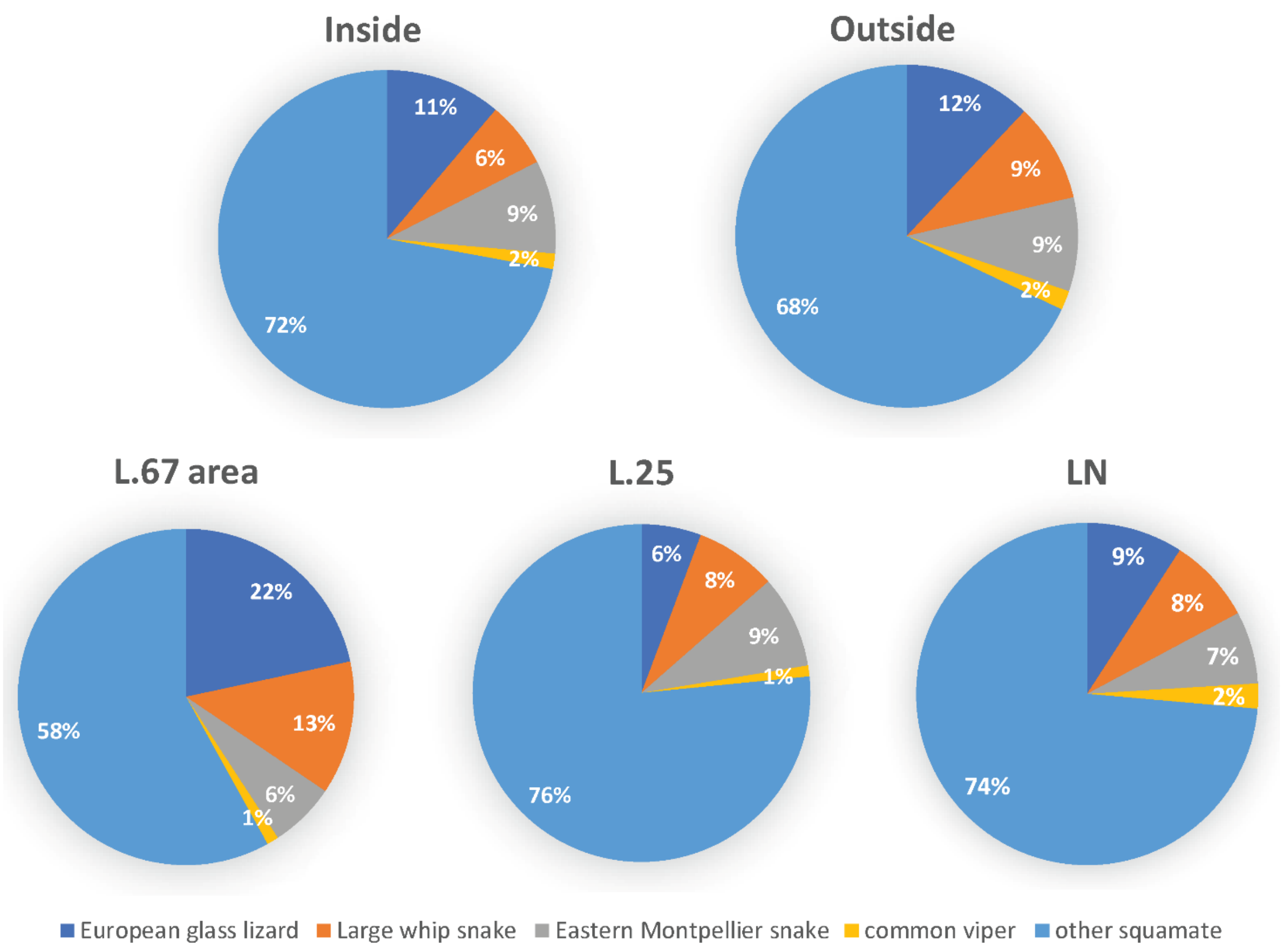

Figure S10. Measurements of vertebra size (centrum length) in the different contexts of the site.

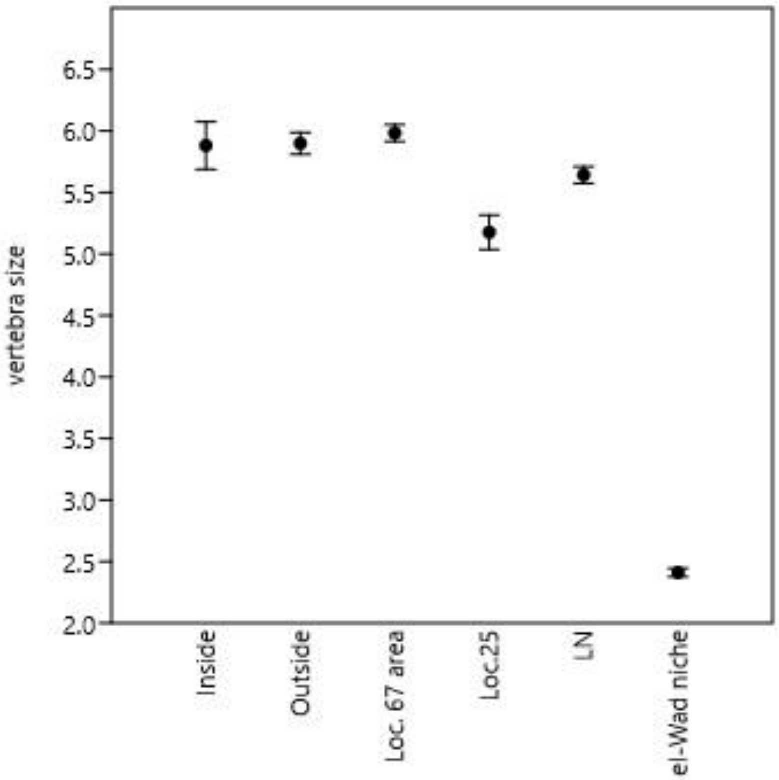

Figure S11. Comparison of digestion intensity (type A+C) among the different contexts of the site and the actualistic assemblages using Adjusted Residuals (AR), data from supplementary table 7.

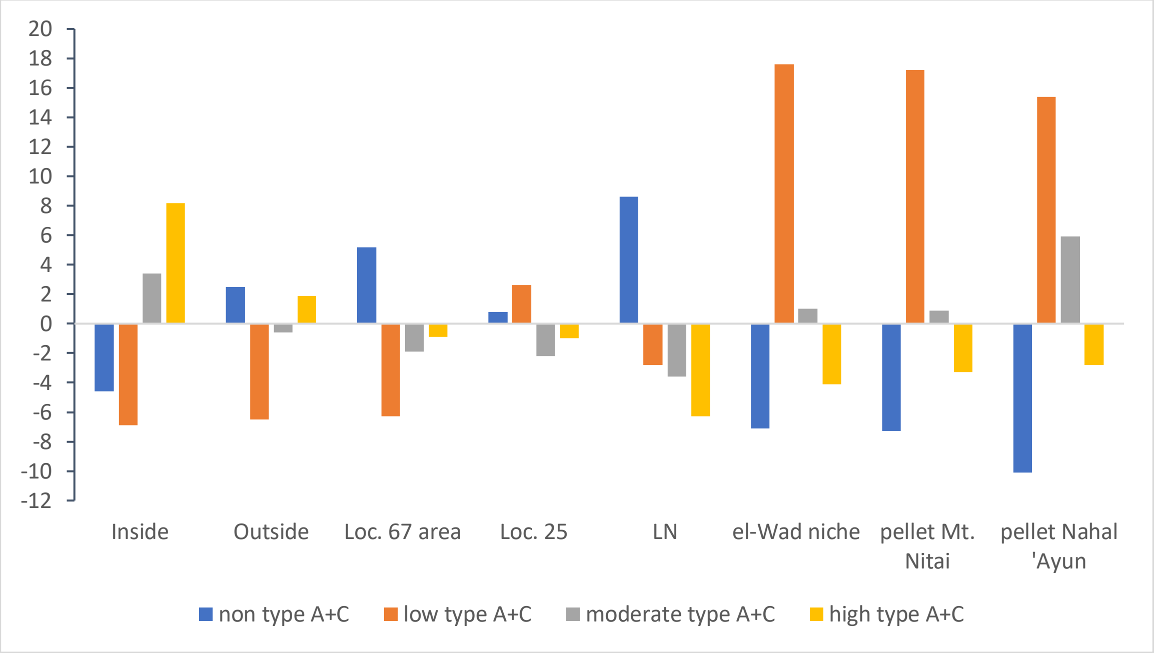

Figure S12. Comparison of digestion intensity (type A+C) among the common species at the site and the actualistic assemblages using Adjusted Residuals (AR), data from supplementary table 7.

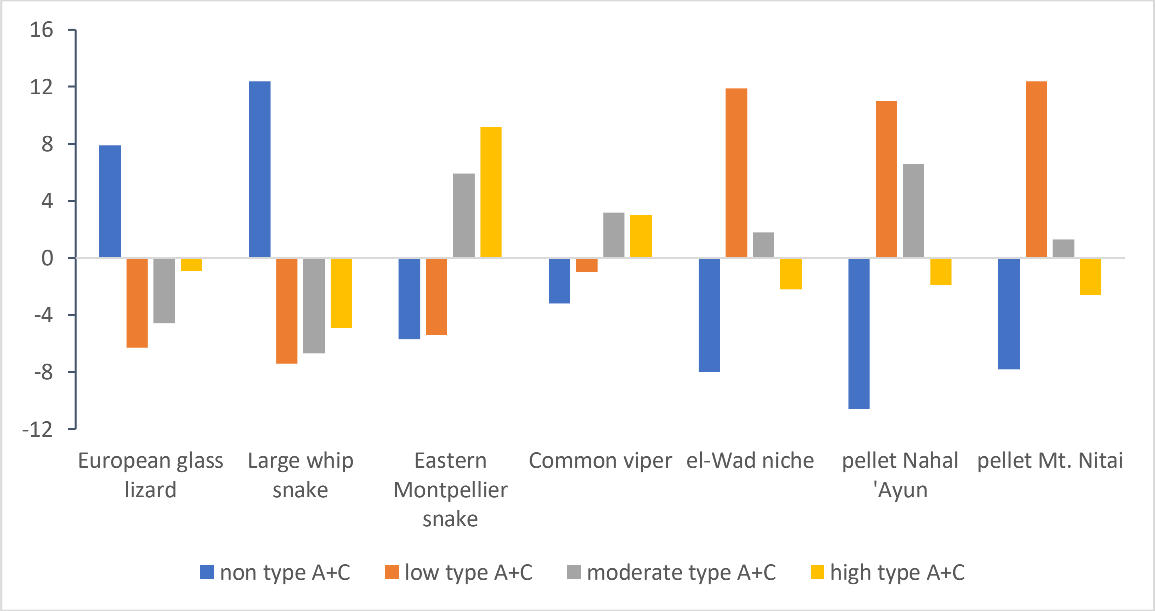

Figure S13. Comparison of erosion intensity among the different contexts of the site, el-Wad niche and the experimental material, data from supplementary table 8.

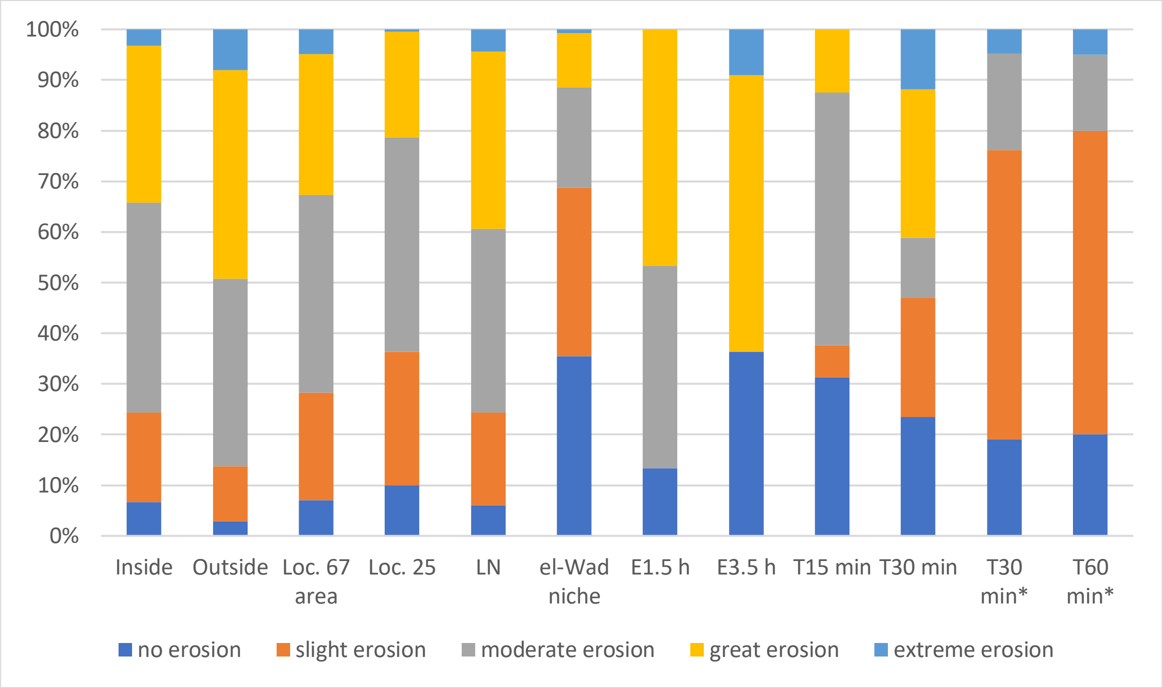

Figure S14. Comparison of cracking intensity among the different contexts of the site and el-Wad niche, data from supplementary table 8.

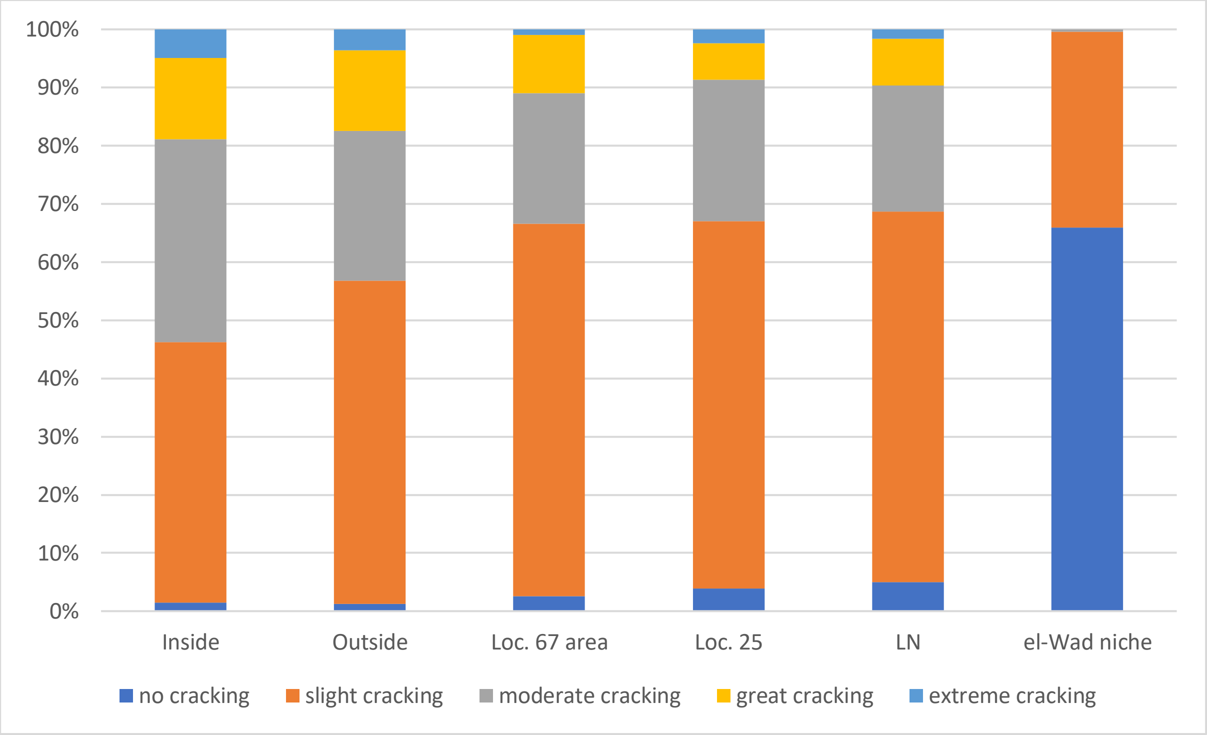

Figure S15. Comparison of flacking intensity among the different contexts of the site and el-Wad niche, data from supplementary table 8.

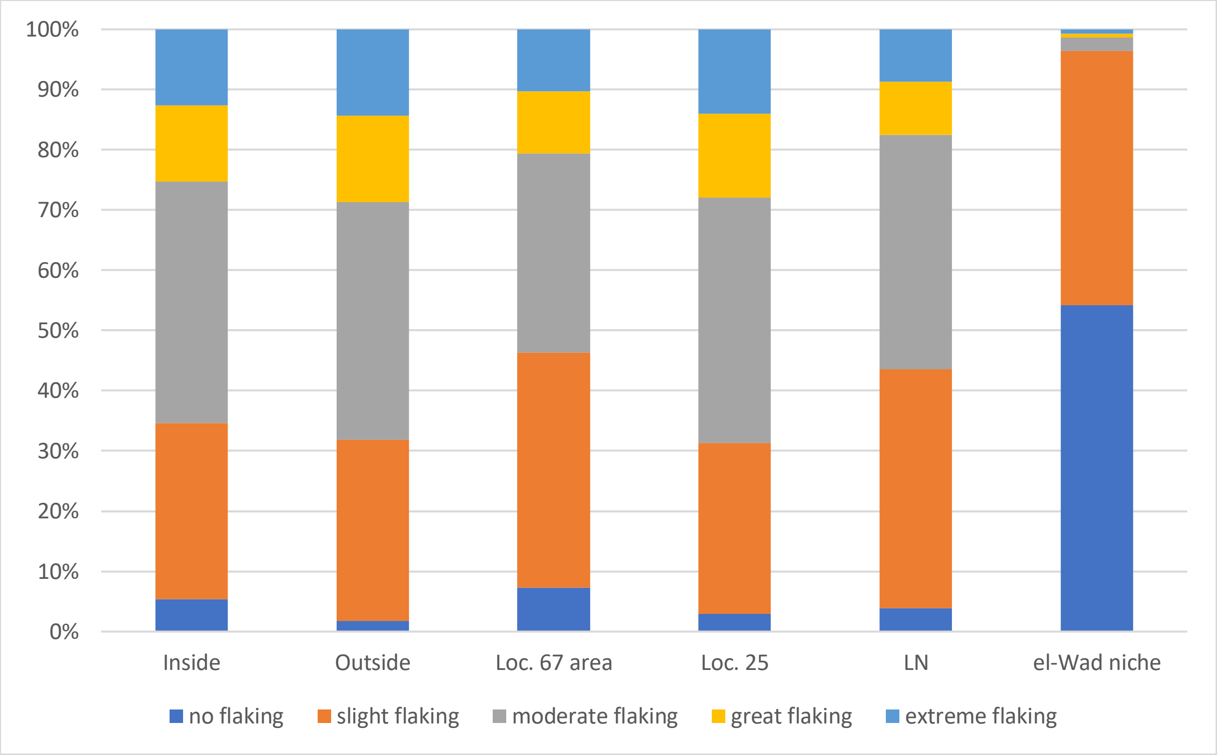

Figure S16. Comparison of protruding parts breakage index among the different contexts of the site, el-Wad niche, owl pellets and the experimental material. Data from supplementary table 8.

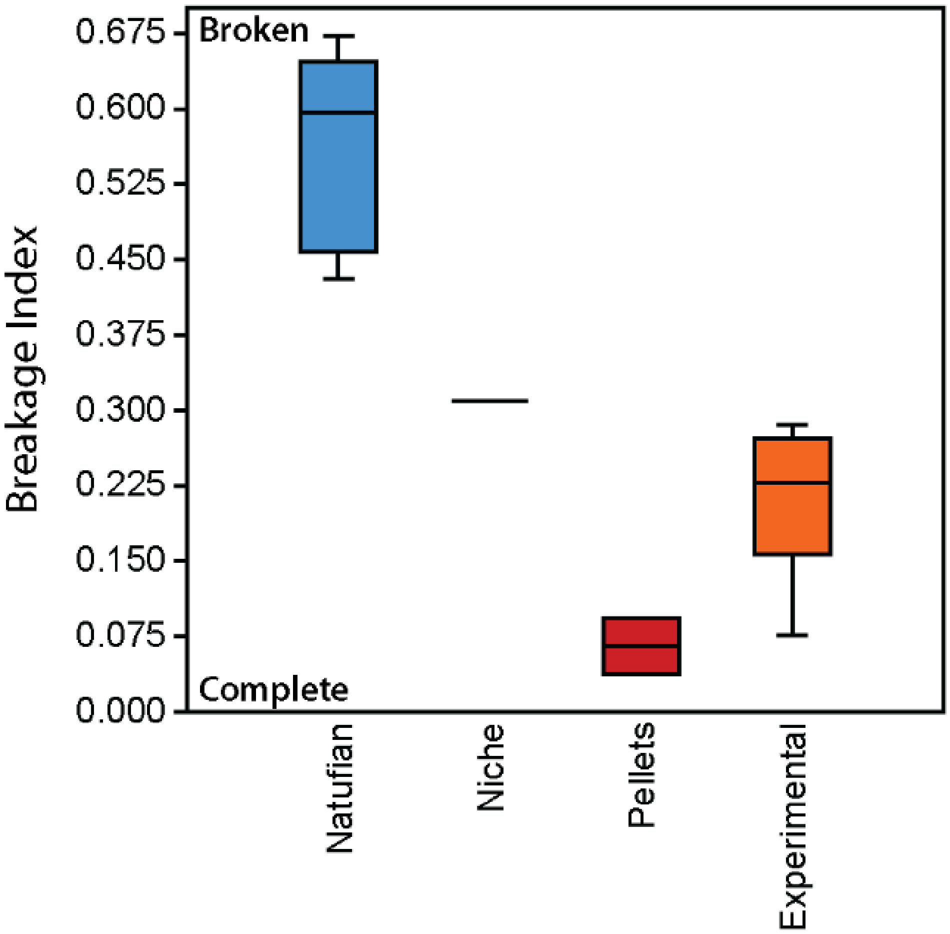

Figure S17. Greatest centrum length measurement (CL) of snake vertebra, ventral view.

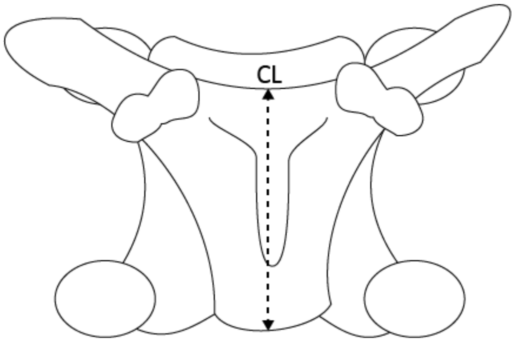

Figure S18. Digestion intensity levels on snake vertebrae.

a) Low digestion level. a- lateral view of a snake trunk vertebra with low digestion levels (x10 magnification); a1- diapophysis with slight and local regular edged perforation of the bone surface (x50 magnification).

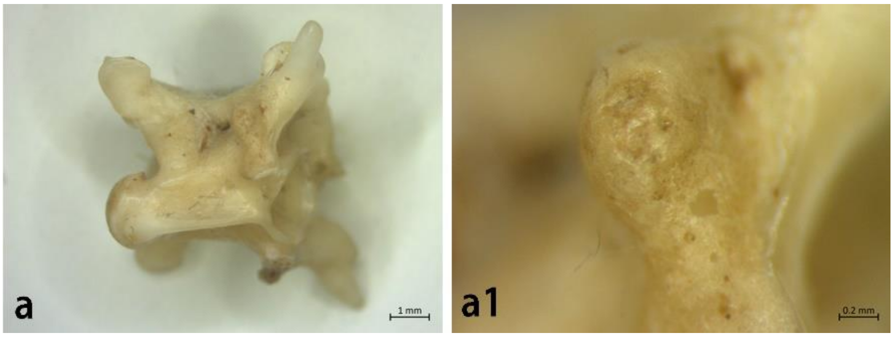

b) Moderate digestion level. a- caudal view of a snake trunk vertebra with moderate digestion levels (x10 magnification); a1- condyle with extensive deeper of the bone surface (x50 magnification).

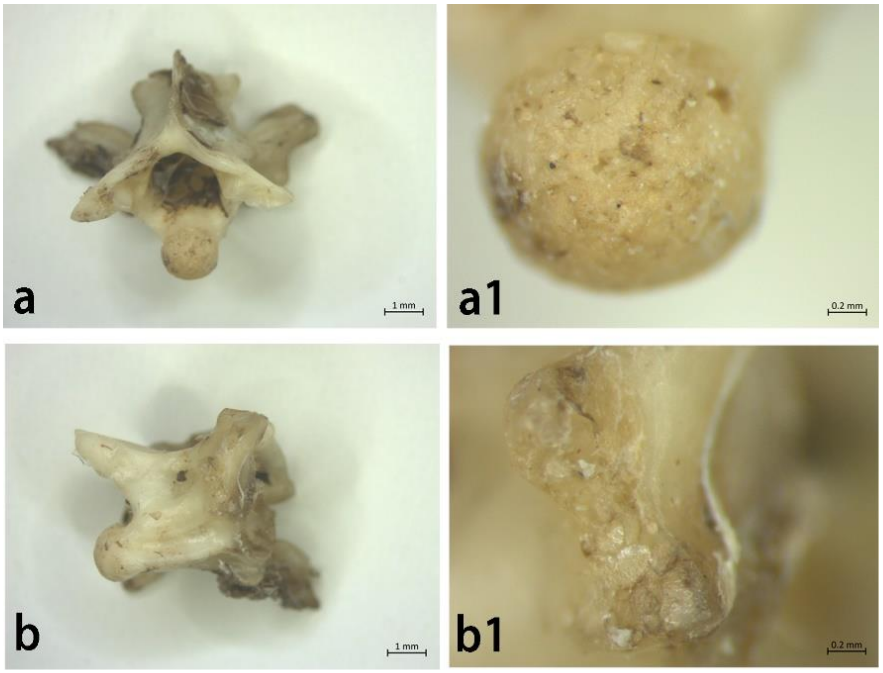

c) High digestion level. a- caudal view of a snake trunk vertebra with high digestion levels (x10 magnification); a1- condyle with extensive deep regular edged

perforation of the bone surface (x25 magnification); b- cranial view of a snake trunk vertebra with high digestion levels (x10 magnification); b1- cotyle with extensive deep regular edged perforation of the bone surface (x25 magnification); c- lateral view of a snake trunk vertebra with high digestion levels (x10 magnification); c1- diapophysis and parapophysis with extensive deep regular edged perforation of the bone surface that may cause some breakage (x25 magnification).

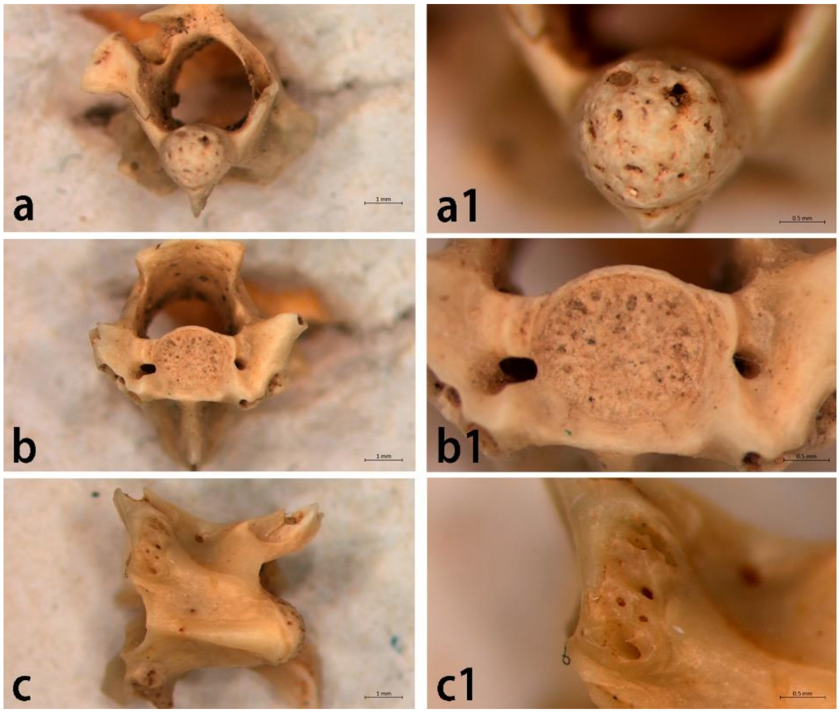

Supplement: Supplementary file 1 — Supplementary information. [file 41598_2020_66301_MOESM1_ESM.pdf]
